# Associations of Green Space and Air Pollution Mixture with Heterogeneous Biological Aging Patterns

**DOI:** 10.1021/EHP.6c00004

**Published:** 2026-09-03

**Authors:** Guoqiang Ma, Jing Xu, Yishu Li, Yuefei Wu, Qian Li, Xuan Cao, Zitong Zhao, Jingyan Gao, Lijun Gao, Lina Yan, Xiaolin Zhang

**Affiliations:** † Hebei Province Key Laboratory of Environment and Human Health, Department of Epidemiology and Statistics, School of Public Health, 12553 Hebei Medical University, Shijiazhuang 050017, China; ‡ Department of Occupational Medicine, The Eighth Hospital of Hebei Province, Shijiazhuang 050021, China

## Abstract

Evidence on associations between green space, air pollution mixture, and heterogeneous biological aging patterns remains limited. We investigated the opposing effects of green space and air pollution mixture on biological aging patterns using an accelerated longitudinal design based on repeated-measures data from 11,760 adults (aged 20–65 years) in a high-pollution Chinese city. Biological age acceleration (BAA) was derived via the Klemera-Doubal method. Group-based trajectory modeling identified three stable BAA profiles, labeled as “slow”, “moderate”, and “accelerated” aging, which were primarily distinguished by overall BAA magnitude rather than divergent slopes over age. Multinomial generalized linear mixed models and weighted quantile sum regression were applied to assess environmental associations. Higher green space (500 m normalized difference vegetation index (NDVI)) was associated with lower odds of membership in the “accelerated aging” trajectory (OR: 0.84; 95% CI: 0.75, 0.95). Even under limited exposure contrast in this high-pollution setting, higher exposures to PM_2.5_, PM_10_, SO_2_, and O_3_ were associated with higher odds of membership in the accelerated aging trajectory. The air pollution mixture showed a consistent positive association (OR: 1.16; 95% CI: 1.08, 1.24), driven primarily by O_3_ and PM_10_. Mediation analysis revealed that 25.0% of the protective association of green space was mediated by reduced pollution exposure. These findings highlight green space as a vital resilience resource, supporting synergistic urban greening and pollution control strategies to promote healthy aging.

## Graphical abstract

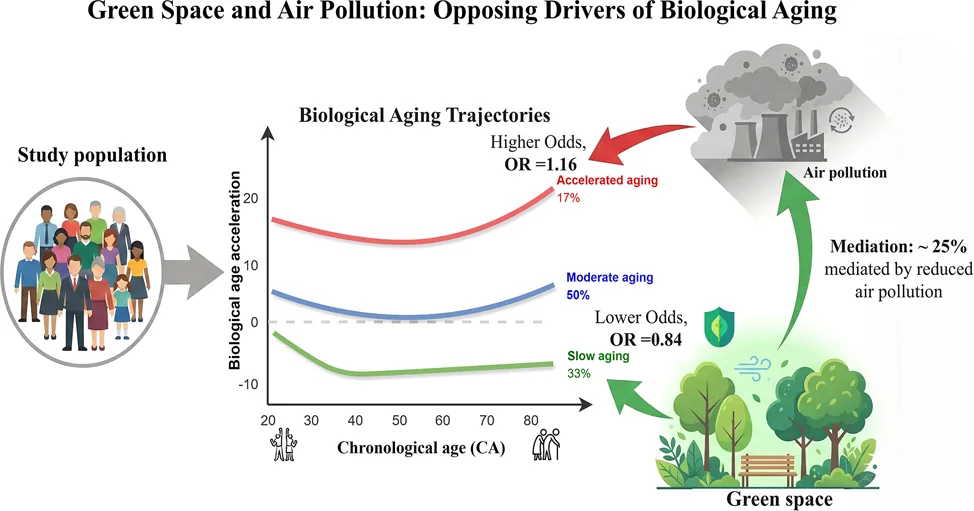

## Introduction

As the global community confronts the dual challenges of population aging and rapid environmental change, elucidating how urban environments shape biological aging has become an imperative in environmental health sciences. According to the World Population Prospects 2024, the global population aged 65 and older is projected to double from approximately 800 million currently to 1.6 billion (16% of the global total) by 2050.^1^ However, extended longevity does not guarantee a commensurate expansion of healthspan. The burden of chronic noncommunicable diseases escalates with advancing age, extending years lived with disability and placing substantial strain on healthcare systems.^2^ Consequently, “healthy aging” has superseded lifespan extension as the central tenet of global public health strategy.^3^ Achieving this goal requires the precise identification and mitigation of modifiable drivers governing the aging process.

Traditionally, aging research has relied on chronological age (CA). While CA merely reflects the passage of time, it overlooks profound interindividual variability in physiological integrity and functional decline.^4^ Aging is fundamentally a dynamic process characterized by the progressive accumulation of damage at the tissue, cellular, and molecular levels.^5,6^ To quantify this interindividual variability, researchers have developed “biological age” (BA) metrics based on clinical biomarkers.^7^ Unlike epigenetic clocks that primarily reflect molecular aging processes, clinical biomarker-based algorithms such as the Klemera-Doubal method (KDM) used in this study characterize systemic physiological integrity and organ function decline, and may be more sensitive to systemic environmental stressors. As a composite measure of physiological wear-and-tear, functional decline, and disease susceptibility, BA serves as a more robust predictor of morbidity and mortality compared to CA.^8,9^ The plasticity of BA offers a unique vantage point to assess the impact of external determinants, including environmental exposures.^10^ Furthermore, recent evidence suggests that biological aging does not follow a uniform linear pattern but rather unfolds through distinct dynamic trajectories across individuals.^11,12^ More recently, conceptual frameworks on biological age clocks have highlighted important limitations of cross-sectional biological age measures, emphasizing that single-time-point assessments cannot distinguish stable age differences from changes in aging patterns over time, thereby underscoring the value of repeated measurements for characterizing biological aging trajectories.^13^ Despite this growing recognition, the environmental factors associated with heterogeneous biological aging trajectories remain poorly understood.

Long-term environmental exposure acts as a critical external force shaping these dynamic trajectories. Urbanization introduces a complex exposome where air pollution and green space represent co-occurring yet opposing environmental forces. Air pollution is a well-established health hazard, linked to aging-related cardiovascular and metabolic pathologies via systemic inflammation, oxidative stress, and DNA damage pathways.^14–16^ Conversely, urban green space constitutes a vital ecosystem resource; higher exposure is consistently associated with reduced mortality and improved physical and mental well-being.^17,18^ The potential mechanisms are multidimensional, encompassing stress reduction, social cohesion, and the promotion of physical activity.^19–21^ The capacity of green space to improve local air quality provides a critical mechanistic entry point for exploring the interplay between these countervailing forces.

Recent studies have made notable strides in linking environmental exposures with biological aging, including investigations into multiplexed environmental factors with multidimensional aging metrics,^22^ urban green and blue space with accelerated aging,^23^ and air pollution and greenspace in relation to biological aging.^24^ Despite this progress, several limitations impede a comprehensive understanding of how the environment modulates aging. First, the preponderance of existing studies relies on cross-sectional designs or single-time aging metrics, providing only “static” associations between exposures and biological age; such snapshot approaches are vulnerable to transient physiological fluctuations and random measurement errors, failing to capture the stable, longitudinal dynamics of aging.^23–26^ Second, environmental factors are frequently assessed in isolation, overlooking the complex “mixture effects” of co-occurring pollutants, which may underestimate the cumulative exposure burden in real-world scenarios.^25,27^ Third, emerging evidence suggests that green space may buffer air pollution-related cardiometabolic, respiratory, cerebrovascular, frailty, and mental health impacts, and that reduced air pollution may mediate some health benefits of greener environments.^28–31^ However, within the current framework of biological aging research, it remains insufficiently explored whether the association between green space and accelerated biological aging is independent of air pollution or is partly explained by lower harmful pollutant exposures.

To systematically address these challenges, this study uses repeated measurements from a longitudinal health examination cohort to identify heterogeneous biological aging patterns at the population level. We constructed an integrated analytical framework combining dynamic aging trajectories, air pollution mixture effects, and mediation analysis. We aim to comprehensively evaluate the complex impacts of long-term exposure to green space and air pollution on longitudinal aging patterns and to quantitatively disentangle the mediating role of air pollution in this relationship.

## Methods

### Study Design and Participants

We employed an accelerated longitudinal (cohort-sequential) design using repeated health examination records from working-age adults. The study was conducted in Shijiazhuang, a major industrial metropolis in Northern China characterized by high population density and substantial industrial pollution burden. This setting provides a critical context for investigating environmental influences on biological aging under high baseline exposure conditions. The initial data set included 18,315 individuals who underwent annual health examinations between January 1, 2021, and December 31, 2023. Participants were eligible if they contributed at least two examination records during the study period, enabling the synthesis of repeated observations across overlapping age ranges. Exclusion criteria were: (1) missing key baseline covariates (e.g., age, sex, workplace); (2) baseline diagnosis of cancer or other severe diseases; and (3) > 50% missingness in biomarkers required for biological age estimation at any single examination. Following these exclusions, missingness for most covariates and biomarkers among retained participants was generally below 10%. Sporadically missing values in the retained sample were imputed using a nonparametric random forest–based algorithm (missForest package) to preserve sample size and reduce information loss associated with complete-case analysis. This approach is well suited for epidemiological data with mixed variable types and complex, potentially nonlinear relationships and has been shown to outperform conventional imputation methods under similar missingness patterns.^32^

To identify robust and generalizable population-level aging patterns, latent aging trajectories were constructed using the full eligible sample of 16,615 individuals spanning the adult age spectrum (age distribution detailed in Figure S2). This strategy maximized age coverage for trajectory derivation within the accelerated longitudinal framework and was used exclusively to derive the population-level BAA trajectory groups. Participants aged >65 years contributed to trajectory derivation but were not included in any exposure-association model. All subsequent association analyses were restricted to 11,760 adults aged 20–65 years (totaling 29,620 observations). This age restriction was applied to ensure the validity of exposure assessment based on workplace addresses, as well as to maintain physiological relevance and methodological consistency. The detailed participant flowchart is presented in Figure S1. The study protocol was approved by the Ethics Committee of Hebei Medical University (No. 2026020), and all participants provided written informed consent.

### Exposure Assessment

The exposure window for all environmental metrics was defined as 2021–2023. We first calculated the annual mean exposure for each year and then averaged these values to derive a three-year mean, representing the cumulative and typical exposure level during the study period. All assessments were based on geocoded workplace addresses. We prioritized workplace exposure to capture environmental conditions during the working population’s active daytime hours. This period is typically characterized by peak traffic-related pollution and increased respiration rates, making workplace environments a biologically relevant proxy for personal exposure, particularly for working-age adults.^33,34^

#### Green Space

Green space exposure was quantified using the normalized difference vegetation index (NDVI). We employed a high-resolution (250 m) monthly NDVI data set covering China, derived from the MOD13Q1 product of the Aqua/Terra-MODIS satellite sensors.^35^ To estimate individual exposure, we calculated the annual mean NDVI within four circular buffers (250, 500, 750, and 1000 m radii) centered on the workplace address. An area-weighted average of all NDVI pixels overlapping the buffer was computed to ensure precise estimation. The 500-m buffer was selected as the primary metric, with other sizes used for sensitivity analyses.^36^

#### Air Pollutants

Long-term (annual mean) exposure to six major air pollutants (PM_2.5_, PM_10_, SO_2_, NO_2_, CO, and O_3_) was assessed using the ChinaHighAirPollutants (CHAP) data set.^37–41^ The CHAP model has been extensively validated across China, including Northern China, with consistently high cross-validated performance reported for major pollutants (cross-validation R^2^ generally exceeding 0.80), supporting its applicability for fine-scale exposure assessment in the present study area. This data set is generated via machine learning fusion of ground-level monitoring, satellite remote sensing, atmospheric reanalysis, and chemical transport model data, yielding high-resolution (1 km × 1 km) gridded data. To refine spatial precision, we employed bilinear interpolation at each geocoded workplace coordinate. By calculating the distance-weighted average of the four nearest grid centroids, this method mitigates spatial boundary effects and captures continuous pollution gradients,^42^ thereby improving the characterization of the ambient environment during working hours.

### Outcome Assessment: Biological Aging Trajectories

The primary outcome was the biological aging trajectory. We applied a three-step analytical framework to identify distinct aging patterns.

#### Step 1: Calculation of BA

We calculated sex-specific BA using the KDM. Briefly, KDM is a multibiomarker algorithm that estimates biological age by integrating age-related clinical biomarkers into a single composite measure of physiological integrity. Detailed formulas and biomarker selection criteria are provided in Methods S1.1.^43,44^ This method has been extensively validated in various Chinese cohorts, confirming its applicability and robustness for our target population.^45–47^ We first calculated the Pearson correlation coefficients between each candidate biomarker and CA. Biomarkers with a correlation of |*r*| > 0.1 were initially selected (Table S1). To address multicollinearity, for pairs of highly correlated biomarkers (|*r*| > 0.7), we prioritized the one with greater clinical relevance or data completeness.^47^ Ultimately, 15 biomarkers were included in the sex-specific models (Table S1). Non-normally distributed biomarkers were log-transformed prior to calculation.^44^

#### Step 2: Calculation of BAA

Because BA is strongly correlated with CA, BAA was calculated as the marginal residuals from a linear mixed-effects model (LMM) regressing BA on CA. This approach removes the population-level aging trend; positive BAA values indicate an older biological age than expected for chronological age.^45,47^ The model yielded an adjusted Intraclass Correlation Coefficient (ICC) of 0.65, indicating that most of the variance in BAA reflects stable interindividual differences rather than transient fluctuations. This longitudinal stability supports the subsequent use of trajectory modeling to identify distinct aging patterns.

#### Step 3: Identification of Heterogeneous Aging Trajectories

GBTM was employed to identify latent subpopulations sharing similar longitudinal BAA patterns.^48^ The resulting trajectory membership was used as the categorical outcome in the exposure-association analyses. Using the lcmm package in R, BAA was modeled as a quadratic function of age to capture potential nonlinear trajectories across the lifespan.^49^ Because BAA reflects deviations from CA, the identified trajectories represent distinct longitudinal patterns of biological aging acceleration relative to the population mean, rather than absolute age-related trends.

By using age as the time scale, we effectively implemented an accelerated longitudinal design. This design allows for the synthesis of overlapping short-term repeated measures from individuals across the entire adult age spectrum into comprehensive life-course aging trajectories.^50^ This approach is particularly advantageous for distinguishing sustained physiological aging patterns from short-term noise. Given the unbalanced longitudinal data structure (individuals with 2 or 3 repeated measures), GBTM effectively incorporates all available observations even for individuals with only two time points to inform the estimation of trajectory shapes, thereby maximizing sample size and statistical power.^51^ We fitted a series of models with 1 to 5 latent classes. The optimal number of groups was determined based on a comprehensive evaluation of statistical fit indices and interpretability, including: lowest Bayesian Information Criterion (BIC), Akaike Information Criterion (AIC), and Integrated Completed Likelihood (ICL); high average posterior probability of assignment (APPA > 0.7); adequate group size (>5% of the sample); and clinical meaningfulness of the trajectory shapes.^12,52–54^ Based on these criteria, the three-group model was selected as optimal. These trajectories were distinct and labeled as “slow aging”, “moderate aging”, and “accelerated aging”. Consequently, aging trajectory was treated as a nominal categorical variable in subsequent analyses.

### Statistical Analysis

Baseline characteristics of the 11,760 participants included in the final analysis were stratified by aging trajectory. Continuous variables, including the three-year mean environmental exposure levels (NDVI and air pollutants) representing long-term typical exposure, are presented as mean ± SD or median (IQR) and were compared using Analysis of Variance (ANOVA) or the Kruskal–Wallis test. Categorical variables are presented as frequency (percentage) and were compared using the Chi-square test.

To examine the association between environmental exposures and aging trajectories, we constructed a series of generalized linear mixed models (GLMMs) with the “slow aging” trajectory serving as the reference group. To account for potential data clustering within workplaces, a random intercept for “workplace” was included in all models. Based on the literature and data availability, the covariate-adjusted models included the following core potential confounders: sex, baseline age (continuous), smoking status (never/ever), alcohol consumption (never/ever), history of chronic noncommunicable diseases (e.g., diabetes, hypertension), and individual-level socioeconomic status, operationalized using household income categories (<100,000, 100,000–200,000, and >200,000 RMB/year).^9^ We additionally fitted unadjusted models that included no fixed-effect covariates, retaining only the exposure and the workplace random intercept, so that estimates before and after covariate adjustment could be compared. Continuous exposure variables were standardized (Z-scores) to facilitate comparison of effect sizes and improve model convergence. We modeled green space exposures (at different buffer sizes) and single air pollutants separately. Results are reported as Odds Ratios (ORs) with 95% Confidence Intervals (CIs) per standard deviation (SD) increase in exposure.

Potential nonlinear relationships were explored using generalized additive mixed models (GAMMs).^55^ Standardized exposure variables were fitted using penalized cubic regression splines, with smoothness automatically determined via restricted maximum likelihood (REML). Nonlinearity was assessed by testing whether the effective degrees of freedom (EDF) of the smooth term was significantly greater than 1. Concentration–response curves with 95% CIs were plotted using the median exposure as the reference.

To evaluate the joint effect of the air pollution mixture on aging trajectories, we applied weighted quantile sum (WQS) regression.^56^ WQS regression summarizes highly correlated pollutants into a single index, assigning each pollutant a non-negative weight to reflect its relative contribution under a prespecified direction of association. Given that standard WQS implementations have limited support for multinomial outcomes, and preliminary analyses indicated no significant differences between the “slow aging” and “moderate aging” trajectories, we dichotomized the outcome into “accelerated” versus “non-accelerated” for this phase and subsequent mechanistic analyses, thereby enhancing model stability and interpretability. To prevent overfitting, the data set was randomly partitioned into a 40% training set (for weight optimization) and a 60% validation set (for effect estimation). Weights for the six pollutants (quartiled) were estimated using 1,000 bootstrap samples. These weights (summing to 1) reflect the relative contribution of each pollutant to the overall mixture effect. The derived WQS index was then entered as a continuous variable into a covariate-adjusted GLMM to estimate the association between the mixture and the odds of accelerated aging.

We further investigated the complex interplay between green space (primary 500-m buffer) and the air pollution mixture (WQS index). First, to test for interaction, we compared nested GLMMs: Model 1 (WQS index + covariates), Model 2 (Model 1 + NDVI main effect), and Model 3 (Model 2 + WQS × NDVI interaction term). Model fit was evaluated using the likelihood ratio test (LRT) and AIC/BIC. The *P* value of the interaction term served as the primary criterion for statistical significance. Upon confirming no significant interaction (*P* > 0.1), we proceeded to formal mediation analysis to explore whether the air pollution mixture (WQS index) mediates the potential protective association between green space (NDVI) and aging trajectories. Using the mediation package in R, we estimated the total effect (TE), natural direct effect (NDE), and natural indirect effect (NIE) via a two-step regression approach (regressing the mediator on exposure, then the outcome on both exposure and mediator), adjusting for the same set of covariates.^57^ Confidence intervals were obtained using quasi-Bayesian approximation (1,000 simulations), and the proportion mediated was calculated as NIE/TE.

The robustness of the findings was systematically assessed across data, model specifications, and potential biases. (1) Robustness of association: We additionally employed linear mixed models (LMM) with continuous BAA as the outcome to verify the consistency of the results with the primary trajectory analysis while accounting for the same set of covariates and random effects. (2) Subgroup analysis: Stratified by sex and age (<40 vs ≥ 40 years) to detect potential effect modification; (3) Exposure categorization: Modeling continuous exposures as quartiles; (4) Temporal stability: Repeating the primary analysis using a one-year lagged exposure window (2020–2022); (5) Spatial scale and alternative greenspace metric: Re-evaluating the primary models using NDVI at alternative buffer sizes (250 m, 750 m, and 1000 m) and employing the Enhanced Vegetation Index (EVI) to ensure the robustness of the findings across different spatial scales and vegetation indices; (6) Missing data: Repeating analyses on the complete-case data set (without imputation); (7) Occupational confounding: additionally adjusting for five broad occupational categories, classified using individual-level occupational information and workplace unit names, to assess potential residual confounding by occupational characteristics related to workplace-based exposure assessment; (8) Outcome model validation: Reidentifying trajectories using growth mixture modeling (GMM), a method with more relaxed assumptions than GBTM;^48^ (9) Mixture model validation: Using quantile g-computation (qgcomp) as an alternative mixture-modeling approach to WQS,^58^ qgcomp estimates the overall mixture effect by jointly shifting all pollutants by one quantile and does not require the assumption of a unidirectional mixture effect. We additionally tested parameter sensitivity by changing WQS quantiles from quartiles to deciles; and (10) Unmeasured confounding: Calculating E-values for significant associations to assess the potential impact of unmeasured confounders.^59^

All statistical analyses were performed using R software (version 4.4.1). Key packages included lme4, lcmm, mgcv, gWQS, mediation, qgcomp, missForest, and Evalue. All tests were two-sided, with *P* value <0.05 considered statistically significant.

## Results

### Characteristics and Environmental Exposure of Participants

The final analytical cohort consisted of 11,760 adults (aged 20–65 years) who contributed a total of 29,620 observations during the study period (mean follow-up frequency: 2.51; 6,100 participants completed three visits). As detailed in Table 1, the overall mean (SD) BA of the population was 42.52 (11.7) years, with a mean BAA of 0.08 (7.73) years. Females accounted for approximately 60% of the sample. The majority of participants were never-smokers (87%) and never-drinkers (74%). Detailed baseline characteristics and the distribution of environmental exposures are provided in Table 1.

**Table 1:** Participant Baseline Characteristics and Three-Year Mean Environmental Exposures by Aging Trajectory^a^

	Aging trajectory			
Characteristics	Slow aging (*N* = 3,832)	Moderate aging (*N* = 5,875)	Accelerated aging (*N* = 2,053)	Overall (*N* = 11,760)
Age (years; mean ± SD)	40.645 ± 8.441	40.327 ± 9.071	41.160 ± 9.498	40.576 ± 8.953
Sex, *n* (%)				
Male	1,335 (34.8)	2,652 (45.2)	685 (33.4)	4,672 (39.7)
Female	2,497 (65.2)	3,223 (54.8)	1,368 (66.6)	7,088 (60.3)
Smoking, *n* (%)				
Never	3,444 (89.9)	5,028 (85.6)	1,765 (86.0)	10,237 (87.0)
Ever	388 (10.1)	847 (14.4)	288 (14.0)	1,523 (13.0)
Drinking, *n* (%)				
Never	2,970 (77.5)	4,153 (70.7)	1,568 (76.4)	8,691 (73.9)
Ever	862 (22.5)	1,722 (29.3)	485 (23.6)	3,069 (26.1)
Household income, RMB/YEAR, *n* (%)				
<100,000	609 (15.9)	1,094 (18.6)	486 (23.7)	2,189 (18.6)
100,000–200,000	2,819 (73.6)	4,112 (70.0)	1,343 (65.4)	8,274 (70.4)
>200,000	404 (10.5)	669 (11.4)	224 (10.9)	1,297 (11.0)
Major diseases, *n* (%)				
Hypertension	123 (3.2)	406 (6.9)	271 (13.2)	800 (6.8)
Diabetes	20 (0.5)	92 (1.6)	142 (6.9)	254 (2.2)
Hypothyroidism	38 (1.0)	47 (0.8)	19 (0.9)	104 (0.9)
Coronary heart disease	22 (0.6)	37 (0.6)	21 (1.0)	80 (0.7)
Hyperlipidemia	20 (0.5)	23 (0.4)	8 (0.4)	51 (0.4)
Biological age				
KDMAge (years; mean ± SD)	34.717 ± 8.560	43.303 ± 9.258	54.860 ± 11.474	42.521 ± 11.679
KDMAge acceleration	–7.254 ± 4.505	1.212 ± 5.001	10.527 ± 4.469	0.080 ± 7.734
Green space (NDVI, mean ± SD)				
250 m	0.288 ± 0.046	0.288 ± 0.046	0.287 ± 0.043	0.288 ± 0.045
500 m	0.289 ± 0.042	0.289 ± 0.042	0.288 ± 0.039	0.289 ± 0.041
750 m	0.290 ± 0.041	0.290 ± 0.041	0.289 ± 0.038	0.290 ± 0.040
1000 m	0.291 ± 0.040	0.291 ± 0.041	0.289 ± 0.038	0.291 ± 0.040
Air pollution (mean ± SD)				
PM_2.5_	47.2 ± 1.4	47.4 ± 1.2	47.7 ± 1.5	47.4 ± 1.3
PM_10_	93.5 ± 1.9	94.2 ± 2.2	94.8 ± 2.0	94.1 ± 2.1
NO_2_	33.1 ± 1.3	33.4 ± 1.2	33.9 ± 1.5	33.4 ± 1.3
SO_2_	8.3 ± 0.9	8.5 ± 0.8	8.8 ± 0.9	8.5 ± 0.9
O_3_	117.2 ± 1.7	117.6 ± 1.6	118.1 ± 1.7	117.5 ± 1.7
CO	0.73 ± 0.04	0.74 ± 0.03	0.75 ± 0.04	0.74 ± 0.04

a Data are presented as mean ± standard deviation (SD) for continuous variables and as number (*n*) with percentage (%) for categorical variables. Environmental exposures were estimated using geocoded workplace addresses, averaged over three years, assigned to participants according to their workplaces, and summarized across participants. Concentrations of PM_2.5_, PM_10_, NO_2_, SO_2_, and O_3_ are expressed in μg/m^3^, whereas CO is expressed in mg/m^3^. Abbreviations: KDMAge, KDM Biological Age; NDVI, Normalized Difference Vegetation Index.

### Identification of Heterogeneous Aging Trajectories

Based on GBTM, we identified three distinct latent aging trajectories characterized by stable stratification across the studied adult age spectrum (Figure 1). Although the model allowed for nonlinear evolution using quadratic terms, the identified subgroups were primarily distinguished by differences in intercepts (overall BAA magnitude at the population level) rather than by divergent slopes over time. The “Slow aging” group (33%, green profile) consistently remained below the population mean, reflecting a sustained state of relative physiological resilience. The “Moderate aging” group (50%, blue profile) followed a trajectory centered around zero, consistent with normative physiological aging. In contrast, the “Accelerated aging” group (17%, red profile) exhibited systematically elevated BAA levels across the entire age range, observable from early adulthood (age 20) through later adulthood. The model selection criteria and fit indices are presented in Table S2.

**Figure 1:**
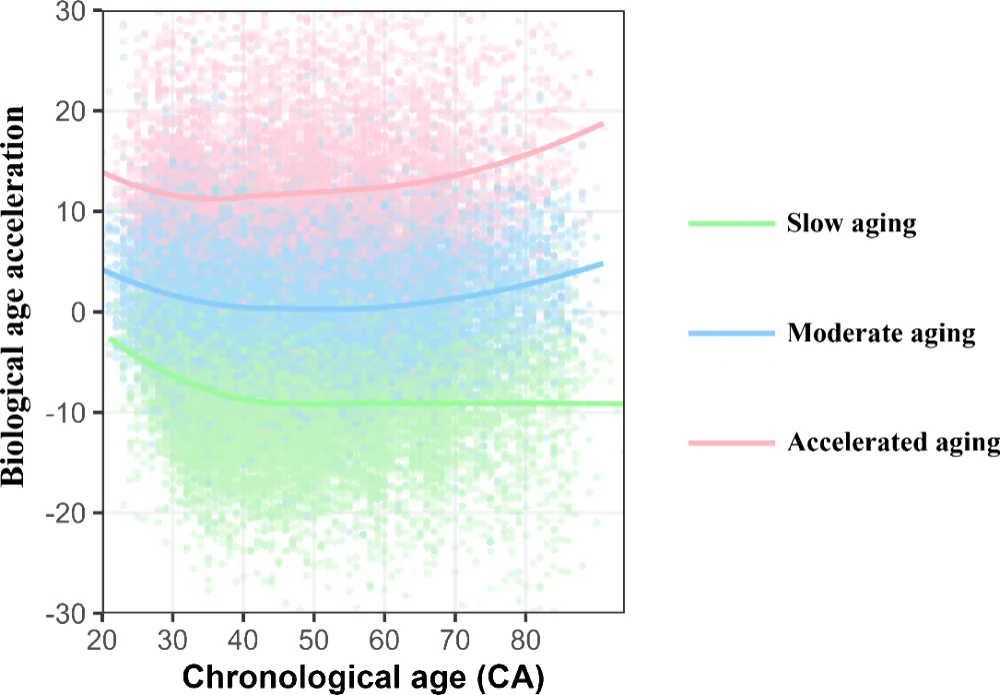
Identified trajectories of BAA across chronological age. The figure displays the three distinct trajectories of BAA identified by GBTM. Each point represents an individual BAA observation, colored according to its assigned trajectory group. The solid lines represent the estimated mean trajectory for each group. The three trajectories were labeled as “slow aging” (*N* = 3,832, 33%; green), “moderate aging” (*N* = 5,875, 50%; blue), and “accelerated aging” (*N* = 2,053, 17%; red).

### Association between Green Space and Aging Trajectories

In fully adjusted models, we observed an inverse association between green space exposure and the odds of membership in the “accelerated aging” trajectory. Among all buffer sizes tested, NDVI within the 500-m buffer exhibited the most robust protective association (Figure 2A). Each 1-SD increase in NDVI (500-m buffer) was associated with 16% lower odds of membership in the “accelerated aging” trajectory relative to the “slow aging” trajectory (OR: 0.84, 95% CI: 0.75–0.95). The corresponding unadjusted NDVI estimates were similar in direction and magnitude, although covariate adjustment modestly strengthened the inverse associations with the “accelerated aging” trajectory (Table S16). GAMM smoothing tests suggested an approximately linear exposure-response relationship (all EDF ≈ 1; Figure 3A–D), with no evidence of meaningful nonlinearity.

**Figure 2:**
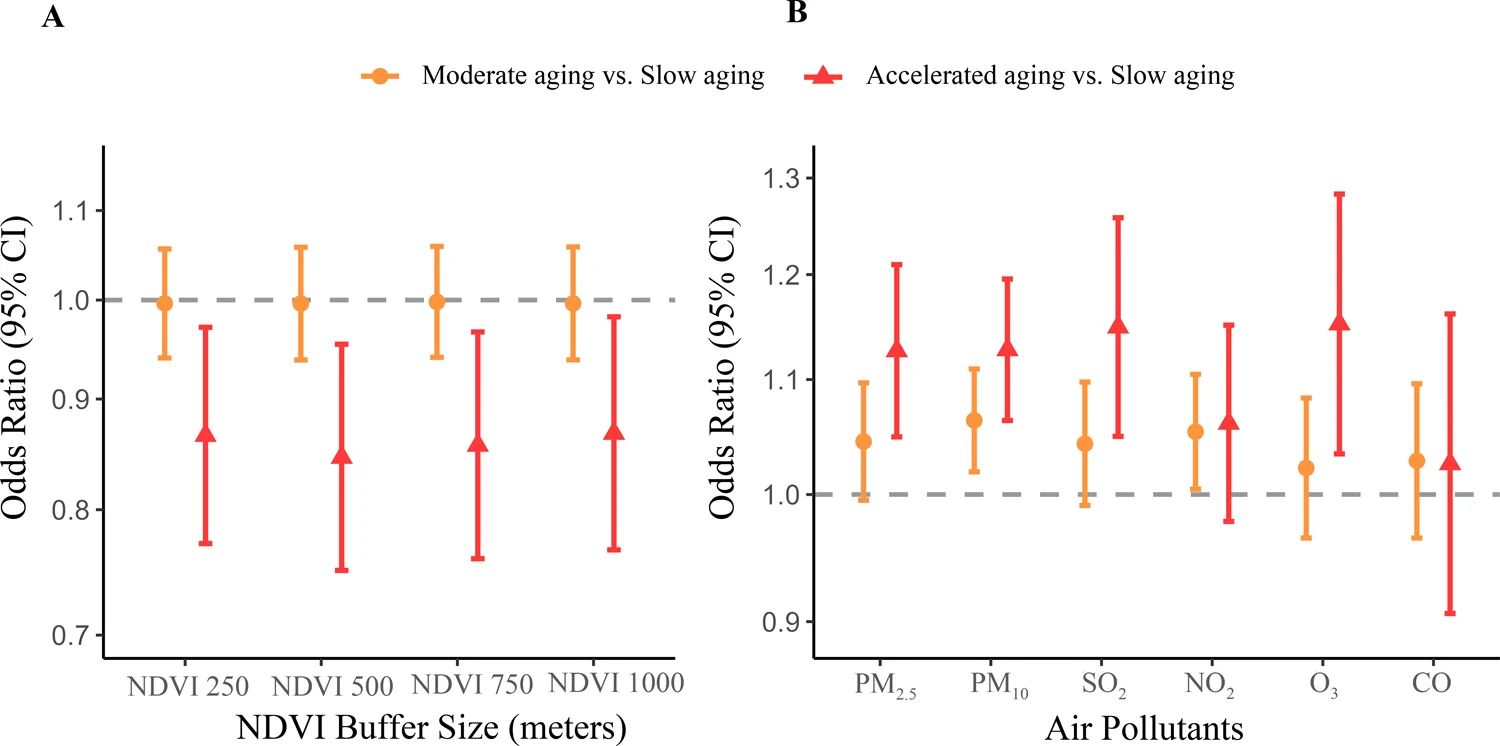
Associations of long-term exposures to green space (A) and single air pollutants (B) with aging trajectories. The figure displays the odds ratios (ORs) and 95% confidence intervals (CIs) for being in the “moderate aging” (orange circles) or “accelerated aging” (red triangles) trajectory, compared to the “slow aging” trajectory, associated with a one standard deviation (SD) increase in each environmental exposure. (A) Associations for green space, assessed by the normalized difference vegetation index (NDVI) across four different buffer sizes (250 m, 500 m, 750 m, and 1000 m). (B) Associations for six individual air pollutants: particulate matter (PM_2.5_, PM_10_), sulfur dioxide (SO_2_), nitrogen dioxide (NO_2_), ozone (O_3_), and carbon monoxide (CO). All estimates were derived from separate multinomial logistic mixed-effects models for each exposure, adjusted for sex, baseline age, smoking status, alcohol consumption, prevalent chronic diseases, and individual-level socioeconomic status, with a random intercept for workplace. The dashed horizontal line indicates the null effect (OR = 1.0).

**Figure 3:**
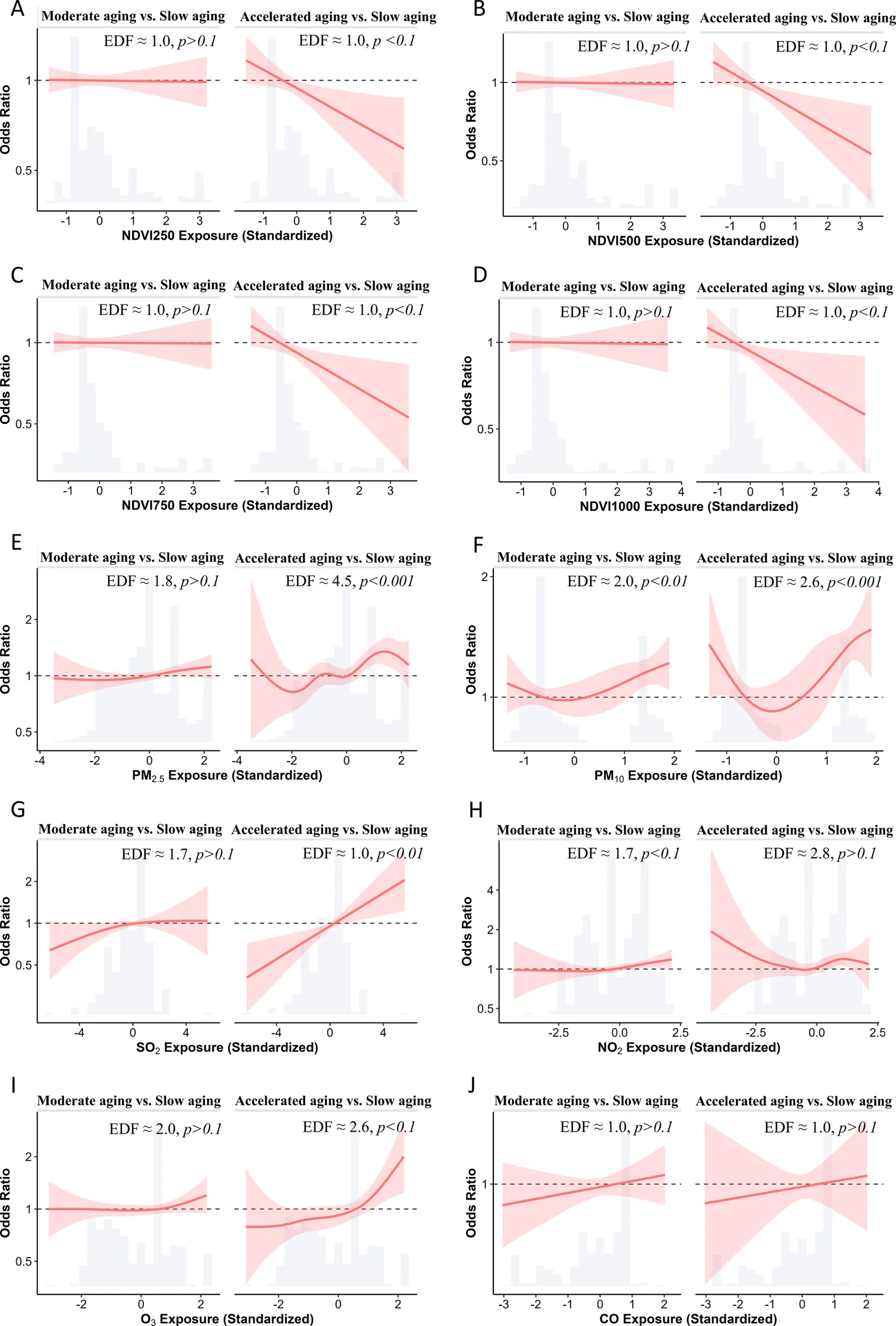
Nonlinear exposure–response relationships between environmental exposures and aging trajectories. The figure displays the concentration–response curves from the fully adjusted generalized additive mixed models (GAMMs). Each panel shows the odds ratio (OR) and 95% confidence interval (CI) for the “moderate vs slow aging” and “accelerated vs slow aging” comparisons across the range of each standardized environmental exposure. The solid red line represents the smoothed estimate derived from a penalized cubic regression spline, with the shaded area representing the 95% CI. The median exposure level was used as the reference (OR = 1.0), indicated by the dashed horizontal line. The histograms in the background represent the distribution of each exposure. Effective degrees of freedom (EDFs) for the smooth term are reported to indicate the degree of nonlinearity; an EDF significantly greater than 1 suggests a nonlinear relationship. The associated *p*-value tests the significance of the smooth term. Panels (A–D) show the associations for NDVI at different buffer sizes. Panels (E–J) show the associations for individual air pollutants.

### Association between Single Air Pollutants and Aging Trajectories

As shown in Figure 2B, multiple air pollutants were associated with higher odds of adverse aging trajectories. Compared to the “slow aging” trajectory, each 1-SD increase in PM_10_ and NO_2_ was associated with higher odds of membership in the “moderate aging” trajectory, with ORs of 1.06 (95% CI: 1.02–1.11) and 1.05 (95% CI: 1.00–1.10), respectively. For the “accelerated aging” trajectory, significant associations were observed for PM_2.5_ (OR: 1.13, 95% CI: 1.05–1.21), PM_10_ (OR: 1.13, 95% CI: 1.06–1.20), SO_2_ (OR: 1.15, 95% CI: 1.05–1.26), and O_3_ (OR: 1.15, 95% CI: 1.03–1.28). SO_2_ and O_3_ exhibited the strongest effect magnitudes. The unadjusted pollutant estimates were also broadly comparable with the covariate-adjusted estimates; adjustment modestly strengthened several associations with the “accelerated aging” trajectory but did not materially change the overall interpretation (Table S16). Inspection of the GAMM splines suggested departures from linearity for PM_2.5_ (EDF ≈ 4.5, *P* < 0.001) and PM_10_ (EDF ≈ 2.6, *P* < 0.001) in the “accelerated aging” versus “slow aging” comparison (Figure 3E,F). However, both pollutants exhibited uneven and multimodal exposure distributions, resulting in limited data support across portions of their exposure ranges; the detailed shapes of these fitted curves should therefore be interpreted cautiously. Across matched percentile-based exposure contrasts, the GAMM- and GLMM-derived estimates were broadly comparable over the exposure ranges with the greatest data support (Table S15). In supplementary analyses based on Gaussian-mixture exposure components, exposure-by-component interaction tests provided no statistical evidence that the linear slopes differed between components for either PM_2.5_ (*P*_interaction_ = 0.731) or PM_10_ (*P*_interaction_ = 0.261) (Table S17). The association for SO_2_ approximated a linear positive trend (EDF ≈ 1.0, *P* < 0.01), whereas O_3_ showed suggestive nonlinearity (EDF ≈ 2.6, *P* < 0.1; Figure 3G,I).

### Joint Effect of Air Pollution Mixture

We applied binary WQS models to estimate the joint effect of the six-pollutant mixture. The WQS analysis demonstrated a significant positive association between the air pollution mixture and the odds of “accelerated aging”. Each 1-SD increase in the WQS index was associated with 16% higher odds of membership in the “accelerated aging” group (OR: 1.16, 95% CI: 1.08–1.24) (Figure 4B). Weights analysis identified O_3_ and PM_10_ as the primary contributors to the mixture effect, with weights of 0.39 and 0.26, respectively (Figure 4A). These findings were corroborated by the binary qgcomp model, which similarly indicated a significant positive association between the mixture and odds of accelerated aging (OR: 1.30, 95% CI: 1.16–1.47), highly consistent with the WQS results. Weight analysis in qgcomp also identified PM_10_ and O_3_ as the dominant positive contributors to the overall mixture association (Figure S12, Table S4).

**Figure 4:**
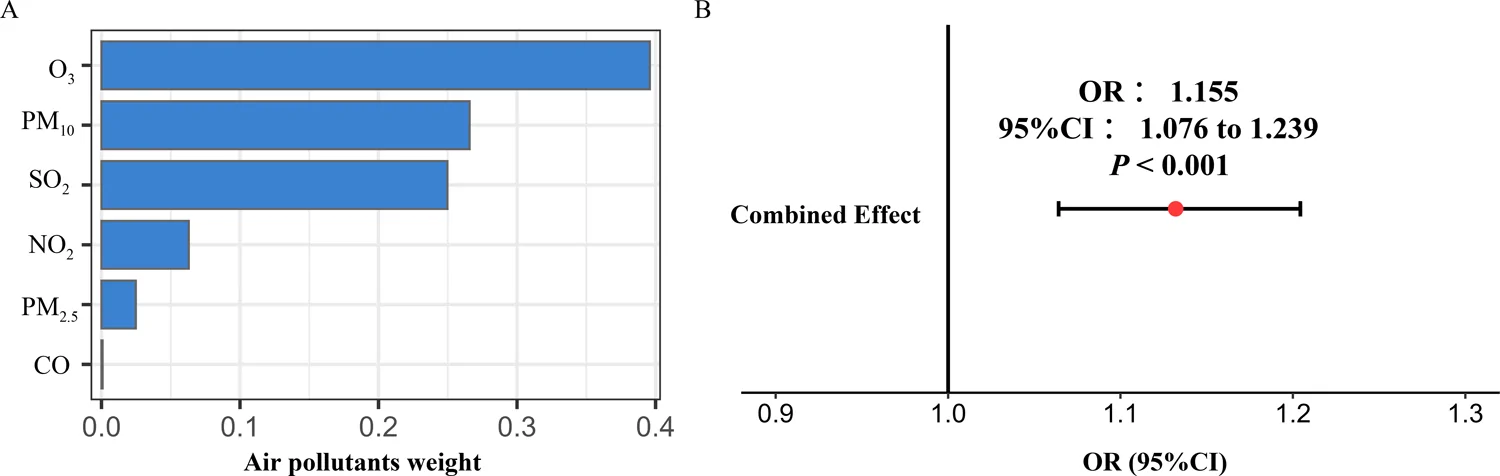
Association of the air pollution mixture with the accelerated aging trajectory from the WQS regression model. Panel (A) shows the weights of the six individual air pollutants contributing to the overall mixture effect. The weights are constrained to sum to 1 and represent the relative importance of each pollutant in the mixture. Panel (B) presents the odds ratio (OR) and 95% confidence interval (CI) for the association between a one standard deviation (SD) increase in the WQS index and the odds of being in the “accelerated aging” trajectory, derived from a fully adjusted logistic mixed-effects model.

### Interaction and Mediation Analysis

We first examined potential interactions between green space (500-m NDVI) and the air pollution mixture (WQS index) (Table S3). No significant multiplicative interaction was detected (*P*_interaction_ = 0.70), suggesting that these factors may influence aging largely independently. Subsequent mediation analysis (Figure 5) revealed a significant total protective effect of green space exposure on the odds of “accelerated aging”. Decomposition of this effect indicated statistically significant natural direct and indirect effects. Specifically, the air pollution mixture partially mediated the association between green space and odds of membership in the “accelerated aging” trajectory, with an estimated proportion mediated of 25% (95% CI: 8.7–67%). This suggests that approximately one-quarter of the protective benefit of green space is attributable to the reduction in ambient air pollution exposure.

**Figure 5:**
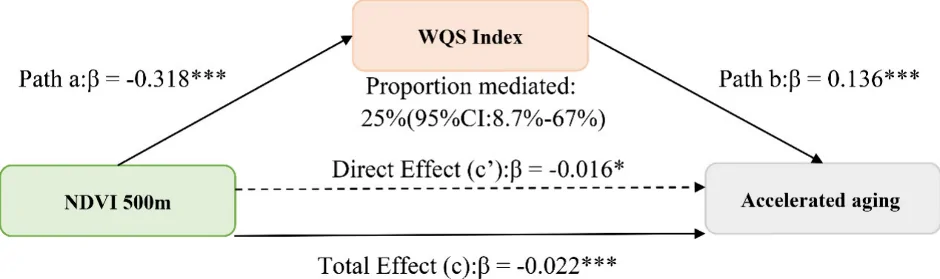
Mediation analysis of the air pollution mixture in the association between green space and the accelerated aging trajectory. The path diagram illustrates the results from the formal mediation analysis, which decomposes the total association between green space (500 m NDVI) and the odds of accelerated aging. The Total Effect (Path c) is partitioned into a Natural Direct Effect (NDE; Path c′) and a Natural Indirect Effect (NIE). The NDE represents the effect of NDVI on the outcome not operating through the air pollution pathway, while the NIE represents the effect transmitted through its influence on the air pollution mixture (WQS index). The final effect estimates (NDE, NIE, and Total Effect) and the coefficients for paths b, c, and c′ are reported on the log-odds scale, representing the change in the log-odds of accelerated aging per one standard deviation (SD) increase in the predictor. The coefficient for Path a is a linear regression coefficient (β), representing the change in the WQS index (in SD units) per one SD increase in NDVI. The proportion mediated was calculated as the ratio of the indirect effect to the total effect (NIE/(NDE + NIE)). Confidence intervals for all effects and the mediated proportion were derived from 1,000 quasi-Bayesian simulations.

### Subgroup Analysis

Stratified analyses by sex and age were conducted to explore potential effect modification (Tables S5–S6). Stratified analyses by sex suggested potential differences in susceptibility. In the comparison of “accelerated aging vs. slow aging”, the association with O_3_ was evident almost exclusively in females (Female OR: 1.25, 95% CI: 1.10–1.42; Male OR: 1.00, 95% CI: 0.88–1.14; *P*_interaction_ = 0.003). Furthermore, although interaction terms did not reach statistical significance (*P*_interaction_ > 0.05), the protective trend of green space and the association estimates for PM_2.5_, SO_2_, and NO_2_ appeared more pronounced in the female subgroup.

Stratified analyses by age also showed variations in associations, with distinct patterns across environmental factors (Tables S7–S8). For green space, the protective association against “accelerated aging” was stronger in younger adults (<40 years), with significant interactions across all buffer sizes (*P*_interaction_: 0.004–0.031). Associations with PM_2.5_, SO_2_, and O_3_ were concentrated in the older group (≥40 years), whereas the associations with PM_10_ and NO_2_ appeared more pronounced in the younger group (<40 years). In addition, the interaction for NO_2_ was statistically significant (*P*_interaction_ = 0.006), with the association largely confined to the younger group (<40 years OR: 1.13; ≥40 years OR: 0.97). A similar pattern was observed for CO (*P*_interaction_ = 0.007).

### Sensitivity Analysis

A series of sensitivity analyses systematically substantiated the robustness of our primary findings (detailed in Supporting Information). First, regarding the outcome definition, LMM with continuous BAA yielded results highly consistent with the primary trajectory analysis. After adjusting for the same set of covariates and random effects, higher exposure to air pollutants (PM_2.5_, PM_10_, NO_2_, SO_2_, and O_3_) remained positively associated with increased BAA, while green space (NDVI-500m) showed a protective association (Figure S7). Second, regarding data and exposure definitions, results remained materially unchanged when using a one-year lagged exposure window (2020–2022), alternative NDVI buffer sizes, an alternative greenness metric (EVI) (Figure S10), or the nonimputed complete data set (Tables S11–S12). Third, regarding model specification, categorizing continuous exposures into quartiles yielded similar dose–response trends (Tables S9–S10). Fourth, to address potential confounding by features of occupation, we further adjusted models for five broad occupational categories. The associations between green space, most air pollutants, and biological aging trajectories were materially unchanged after this additional adjustment (Figure S13). The association for NO_2_ was modestly attenuated and no longer reached statistical significance, while the overall pattern and interpretation of the primary findings remained consistent. Fifth, validation of the outcome model using growth mixture modeling (GMM), which assumes more relaxed parameters, identified three aging trajectories with shapes highly similar to those from GBTM. The concordance between GBTM and GMM classifications was excellent (unweighted Cohen’s Kappa = 0.95), indicating that our trajectory groups were robust to model assumptions (Table S13). Sixth, rigorous validation of the mixture analysis confirmed our results. Increasing the WQS quantile resolution from quartiles to deciles did not alter the direction or magnitude of associations (Table S4). Moreover, exploratory multinomial qgcomp analysis revealed a gradient of higher odds, where the mixture was associated with both “moderate aging” (OR: 1.13, 95% CI: 1.03–1.26) and “accelerated aging” (OR: 1.39, 95% CI: 1.20–1.60) compared to “slow aging” (Table S4). Finally, E-value calculations for significant associations ranged from 1.18 to 1.39 (Table S14), values consistent with typical observational environmental studies, suggesting that unmeasured confounding is unlikely to fully explain the observed associations.

## Discussion

In this study, we identified three distinct biological aging trajectories (“slow aging”, “moderate aging”, and “accelerated aging”) characterized by significant heterogeneity. Recent studies have linked multiplexed environmental factors, green/blue space and air pollution with biological aging metrics.^22–24^ Building on this evidence, this study extends prior work by jointly evaluating workplace-based green space and air pollution mixtures in relation to heterogeneous biological aging trajectories, while further exploring the potential mediating role of air pollution. Across multiple analytical approaches, including concentration–response modeling and sensitivity analyses using LMM, we observed consistent associations between environmental exposures and aging trajectories. Higher long-term exposure to green space was associated with lower odds of membership in the “accelerated aging” trajectory (OR: 0.84 per SD increase in NDVI-500m), whereas elevated exposure to air pollutants (PM_2.5_, PM_10_, NO_2_, SO_2_, and O_3_) corresponded to higher odds of membership. Despite the relatively low spatial variation of air pollutants within the study area, significant associations were observed. This suggests that in a high-pollution background setting, population-level biological aging processes may be sensitive to even modest differences in cumulative exposure burden. Mixture analysis further revealed a positive association between the overall air pollution burden and the odds of accelerated aging, with PM_10_ and O_3_ identified as the dominant mixture contributors. Notably, mediation analysis indicated that approximately 25% of the protective association between green space and biological aging trajectories may be statistically mediated by lower air pollution levels in greener environments. However, since environmental exposures were assessed concurrently, these mediation findings should be interpreted as associative patterns consistent with a mediation hypothesis rather than as evidence of definitive causal pathways.

We observed that individuals with higher long-term green space exposure were more likely to exhibit a healthier aging pattern. This finding is consistent with recent evidence linking urban green/blue spaces with healthier biological aging metrics, as well as previous cross-sectional studies; for instance, a study of 156,690 participants reported a protective association between green space and epigenetic age acceleration,^24^ and another cohort study linked urban greenery to reduced KDM biological age.^23^ However, by identifying distinct aging patterns based on repeated measurements, our study extends these “static” associations to a dynamic longitudinal perspective. Rather than reflecting transient physiological improvements, our findings suggest that green space may contribute to sustained physiological resilience against biological aging. In urban environments characterized by persistently high pollution burdens, green space may therefore function as a vital resilience resource. The potential biological mechanisms underlying this protection are likely multifaceted. First, mediation analysis provides quantitative support for an indirect pathway whereby the protective association of green space is partly explained by lower ambient air pollution levels. Second, the predominant direct effect likely stems from the psychophysiological restorative functions of nature. Evidence suggests that contact with natural environments can alleviate chronic stress, improve mental health, and downregulate the overactivation of the hypothalamic-pituitary-adrenal (HPA) axis, thereby mitigating systemic inflammation and oxidative stress, processes intimately linked to biological aging.^17,18,20^ Additionally, green spaces may promote physical activity, a well-recognized behavioral pathway contributing to slower biological aging. While physical activity could not be directly assessed in this study, it likely operates as a complementary mechanism alongside stress alleviation and air pollution mitigation.

Regarding air pollution, this study substantiates and extends prior evidence by showing that both individual pollutants and the overall air pollution mixture were associated with heterogeneous BAA patterns. The single-pollutant results were broadly consistent with prior findings in European and Chinese populations showing that exposure to PM_2.5_, PM_10_, NO_2_, and O_3_ is associated with accelerated biological aging.^22,24,25^ Notably, these associations were detected despite relatively limited interindividual variation in air pollutant concentrations, likely reflecting three-year exposure averaging, clustering of participants within shared workplaces, and the uniformly elevated pollution background that generally exceeded WHO air quality guideline values. Although such constrained exposure contrast requires cautious interpretation of the exposure-response magnitude, the consistent associations observed across analyses suggest that biological aging may remain sensitive to even modest differences in pollution exposure. This observation aligns with growing evidence that the adverse health effects of air pollution persist without a clear safe threshold, leaving physiological systems vulnerable to even small increments in exposure under conditions of chronic environmental stress. Air pollution may induce DNA damage, telomere attrition, and mitochondrial dysfunction via oxidative stress and chronic inflammatory responses, thereby accelerating cellular senescence and systemic physiological decline.^14–16^ Furthermore, alterations in DNA methylation and epigenetic aging markers linked to multipollutant exposure provide molecular plausibility for these long-term associations.^60^ In this context, our KDM-based outcome should be interpreted as a clinically grounded measure of systemic physiological aging rather than as a direct molecular clock. Although KDM-BA does not directly capture DNA methylation clocks or other omics-based markers and is constrained by available routine clinical measurements, it is well suited to large-scale health examination cohorts and captures multisystem physiological integrity relevant to inflammation- and oxidative stress-related environmental pathways. To evaluate environmental correlates of this systemic aging process more comprehensively, our application of WQS and qgcomp models captured the complexity of real-world joint exposures and consistently indicated an adverse mixture association. Both models identified PM_10_ and O_3_ as the highest-weighted components, further implicating oxidative stress as a core pathway driving pollution-related accelerated aging. Although the GAMM splines suggested departures from linearity for PM_2.5_ and PM_10_, their uneven and multimodal exposure distributions limited data support across portions of the exposure ranges; the detailed curve shapes should therefore not be regarded as conclusive evidence of U-shaped or threshold relationships. The broadly comparable GAMM and GLMM estimates across data-supported contrasts, together with interaction tests that provided no statistical evidence of slope heterogeneity between exposure components, suggest that these local spline fluctuations did not materially alter the overall interpretation of the primary GLMM results.

Beyond delineating independent effects, we found that approximately 25% of the protective association between green space and healthier aging patterns was statistically mediated by mitigation of air pollution exposure. This suggests that green space may function as a dual-purpose environmental resource: acting as a “passive barrier” to pollutants while engaging in “active restoration”.^61^ The coexistence of direct and indirect effects provides a novel perspective on the synergistic potential of these environments. Our findings suggest that particularly in high-pollution scenarios, green space exerts a substantial indirect protective effect by facilitating pollutant deposition and dispersion.^62^ From a public health perspective, increasing urban greenery may represent a robust strategy with complementary benefits even in densely populated, high-pollution urban settings. Our findings provide supportive evidence for synergistic urban governance approaches, in which green infrastructure development is integrated with air quality management to promote healthier aging.

Our subgroup analyses revealed significant heterogeneity in the associations between environmental exposures and biological aging, delineating a complex “population susceptibility profile”. Stratified analyses by sex indicated a heightened environmental sensitivity among females. The association between O_3_ and accelerated aging was observed almost exclusively in women (*P*_interaction_ = 0.003), and adverse associations with PM_2.5_, SO_2_, and NO_2_ also appeared more pronounced in the female subgroup. This aligns with emerging consensus in environmental epidemiology that women may be more vulnerable to specific environmental stressors.^63^ Potential mechanisms for this susceptibility are likely multifaceted. While this observational study cannot verify specific biological pathways, previous literature suggests that hormonal fluctuations, particularly estrogens, may modulate inflammatory and oxidative stress pathways, thereby altering physiological responses to pollutants.^64^ Behaviorally, sex-based differences in commuting patterns and leisure activities may lead to distinct exposure profiles.

Stratified analyses by age further identified age as a critical effect modifier. Although the protective association of green space reached statistical significance only in the older group (≥40 years), both interaction tests and point estimates consistently indicated a stronger protective trend in younger adults (<40 years). This pattern, characterized by a stronger effect size but lower statistical significance, likely reflects limited statistical power due to the smaller sample size of the younger subgroup. Synthesizing these evidence, we postulate that the protective role of green space may be particularly pivotal in younger adulthood. This implies that accumulating an environmental “dividend” (e.g., greater contact with nature) during younger years may help shape more favorable aging-related patterns and bolster physiological resilience against future environmental challenges.^65^ However, this life-course–relevant hypothesis warrants further validation through larger cohorts with extended age spans.^66^ Associations of PM_2.5_, SO_2_, and O_3_ with accelerated aging were concentrated in middle-aged and older adults (≥40 years), likely reflecting the combined impact of cumulative damage and declining physiological reserve. Associations of PM_10_ and NO_2_ with accelerated aging were more prominent in the younger group (<40 years). While the exact mechanism remains to be elucidated, this may point to unique exposure patterns (e.g., traffic-related NO_2_ exposure during commuting) or distinct physiological response pathways in younger individuals, necessitating further investigation. Overall, these findings emphasize that future environmental health research and interventions must move beyond “average effects” to account for these critical modifiers, thereby enabling more precise risk assessment and protection strategies.

Against the backdrop of rapid urbanization and global population aging, our findings have important public health relevance. This study provides supportive evidence that urban environmental interventions may represent a feasible strategy for promoting healthy aging. Specifically, the expansion of green space and air pollution control should be viewed as synergistic rather than isolated environmental strategies. Particularly in cities with persistently high pollution levels, urban greenery not only confers direct psychophysiological benefits but also plays a substantial indirect protective role by mitigating local exposure to pollutants. Collectively, these findings suggest that “greening” and “pollution reduction” may be regarded as complementary public health goals. Incorporating green infrastructure into urban planning while simultaneously strengthening air quality management may yield substantial benefits for promoting healthier aging in urban populations.

This study possesses several strengths. First, we adopted an accelerated longitudinal (cohort-sequential) design and leveraged repeated measurements across overlapping age groups to identify heterogeneous biological aging patterns at the population level. Traditional cross-sectional studies based on a single assessment are vulnerable to random measurement error and short-term biological variability. In contrast, our repeated-measures design increased the signal-to-noise ratio of biological aging indicators, and the application of group-based trajectory modeling facilitated the identification of latent, relatively stable aging patterns within the population. Second, within a typical high-density urban setting with persistently elevated pollution levels, we systematically evaluated the independent, joint, and interactive effects of green space and air pollution mixture on biological aging. Third, the robustness of our findings was rigorously assessed using complementary modeling strategies, including LMM with continuous biological age acceleration as the outcome, which yielded results consistent with the primary trajectory-based analyses. Several limitations should be acknowledged. First, participants with missing key covariates or extensive biomarker missingness were excluded before imputation. Although imputation addressed sporadic missingness within the retained sample and complete-case analyses yielded consistent findings, potential selection bias arising from these preimputation exclusions cannot be ruled out. Second, despite adjusting for major covariates, residual confounding cannot be entirely excluded. However, the calculated E-values (1.18–1.39) suggest that an unmeasured confounder would need to have substantially stronger associations with both exposure and outcome than those observed to fully explain the findings. Third, exposure assessment relied on workplace addresses. While lacking residential data introduces nondifferential misclassification, workplace environments capture the critical active daytime window characterized by higher physical activity and respiration rates. Such random error generally biases associations toward the null, implying that the true effects may be even stronger. Therefore, our findings complement residential-based evidence and highlight the need to improve environmental quality in occupational settings. Furthermore, workplace locations may correlate with certain features of occupation. Although the study population was predominantly composed of indoor and office-based workers, with limited representation of occupations involving substantial occupational hazard exposures or prolonged outdoor work, and we further adjusted for broad occupational categories to account for differences in job type and potential exposure to hazards, residual confounding related to unmeasured occupational characteristics, such as work complexity and psychosocial work environments, cannot be completely excluded. Fourth, although the accelerated longitudinal design enabled the estimation of population-level aging patterns across age groups, individual follow-up durations were relatively short. Future studies with extended follow-up periods are warranted to further capture the long-term intraindividual dynamics of environmental influences on aging.

## Conclusion

In summary, this study, employing an accelerated longitudinal design on a cohort of working-age adults in a typical high-pollution urban setting in China, provides robust evidence regarding environmental correlates of biological aging. We identified relatively stable biological aging patterns within the population, showing that higher long-term green space exposure was associated with healthier aging patterns, whereas exposure to the air pollution mixture was associated with higher odds of accelerated aging. Green space appears to function as a vital resilience resource, potentially operating through two complementary pathways: direct psychophysiological restoration and indirect mitigation of air pollution exposure. Collectively, these findings underscore the critical importance of adopting a multiexposure perspective and provide a solid scientific basis for synergistic urban interventions integrating green infrastructure with pollution control to promote healthy aging in rapidly urbanizing populations.

## Supplementary Material



## Data Availability

The individual-level health examination data generated in this study are not publicly available due to privacy and ethical restrictions but may be made available by the corresponding author upon reasonable request.

## Notes

Additional methodological details; Pearson’s correlation coefficients, model fit statistics, nested models, sensitivity analyses, sex- and age-stratified associations, quantified green space and air pollutant exposures, cross-classifications, *E* values, and quantitative comparisons (Tables S1–S17); flow diagram, distribution and correlation data, model fit data, temporal trends, Spearman’s correlation coefficients, high-resolution spatial distribution, associations between environmental exposure and biological age, sensitivity analyses, pollutant weights, associations of green space and air pollutants with aging trajectories, and comparison of GAMM and GLMM with rug plots (Figures S1–S14) (PDF)

## References

[1] United Nations Department for Economic and Social Affairs . World Population Prospects 2024: Summary of Results; United Nations: New York, 2025.

[2] Licher S., Heshmatollah A., van der Willik K. D., Stricker B. H. C., Ruiter R., de Roos E. W., Lahousse L., Koudstaal P. J., Hofman A., Fani L., Brusselle G. G. O., Bos D., Arshi B., Kavousi M., Leening M. J. G., Ikram M. K., Ikram M. A. (2019). Lifetime risk and
multimorbidity
of non-communicable diseases and disease-free life expectancy in the
general population: A population-based cohort study. PLOS Medicine.

[3] Wang H., Qin D., Fang L., Liu H., Song P. (2024). Addressing Healthy
Aging in China: Practices and Prospects. BioScience
Trends.

[4] Moqri M., Herzog C., Poganik J. R., Justice J., Belsky D. W., Higgins-Chen A., Moskalev A., Fuellen G., Cohen A. A., Bautmans I., Widschwendter M., Ding J., Fleming A., Mannick J., Han J.-D. J., Zhavoronkov A., Barzilai N., Kaeberlein M., Cummings S., Kennedy B. K., Ferrucci L., Horvath S., Verdin E., Maier A. B., Snyder M. P., Sebastiano V., Gladyshev V. N. (2023). Biomarkers
of Aging for the Identification and Evaluation of Longevity Interventions. Cell.

[5] López-Otín C., Blasco M. A., Partridge L., Serrano M., Kroemer G. (2013). The Hallmarks
of Aging. Cell.

[6] Musi, N. ; Hornsby, P. J. Handbook of the Biology of Aging; Elsevier, 2021; 10.1016/B978-0-12-815962-0.00020-2.

[7] Levine M. E. (2013). Modeling
the Rate of Senescence: Can Estimated Biological Age Predict Mortality
More Accurately than Chronological Age?. J.
Gerontol A Biol. Sci. Med. Sci..

[8] Faul J. D., Kim J. K., Levine M. E., Thyagarajan B., Weir D. R., Crimmins E. M. (2023). Epigenetic-Based
Age Acceleration
in a Representative Sample of Older Americans: Associations with Aging-Related
Morbidity and Mortality. Proc. Natl. Acad. Sci.
U. S. A..

[9] Levine M. E., Lu A. T., Quach A., Chen B. H., Assimes T. L., Bandinelli S., Hou L., Baccarelli A. A., Stewart J. D., Li Y., Whitsel E. A., Wilson J. G., Reiner A. P., Aviv A., Lohman K., Liu Y., Ferrucci L., Horvath S. (2018). An Epigenetic Biomarker of Aging
for Lifespan and Healthspan. Aging.

[10] Pusparum M., Thas O., Beck S., Ecker S., Ertaylan G. (2025). From Ageing
Clocks to Human Digital Twins in Personalising Healthcare through
Biological Age Analysis. npj Digit. Med..

[11] Li X., Ploner A., Wang Y., Magnusson P. K., Reynolds C., Finkel D., Pedersen N. L., Jylhävä J., Hägg S. (2020). Longitudinal
Trajectories, Correlations and Mortality
Associations of Nine Biological Ages across 20-Years Follow-Up. eLife.

[12] Wang S., Li W., Li S., Tu H., Jia J., Zhao W., Xu A., Xu W., Tsai M. K., Chu D. T.-W., Wen C. P., Wu X. (2023). Association between Plant-Based Dietary Pattern and Biological Aging
Trajectory in a Large Prospective Cohort. BMC
Medicine.

[13] Zalesky A., Wen J., Tian Y. E. (2026). From Whole-Body to Organ-Specific Biological Age Clocks. Nat. Aging.

[14] Mesnage R. (2025). Environmental
Health Is Overlooked in Longevity Research. Antioxidants.

[15] Sullivan J., Sorensen C. (2023). Protecting Populations from the Health Harms of Air
Pollution. BMJ..

[16] Xu H., Huang K., Zhang B., Yang H., Wang J., Li X., Meng X., Chen R., Zhang X. (2025). Associations of Outdoor
Ozone Concentration with Thyroid Function and the Mediated Role of
Serum Metabolites: A Panel Study of Healthy Children. Journal of Hazardous Materials.

[17] Rojas-Rueda D., Nieuwenhuijsen M. J., Gascon M., Perez-Leon D., Mudu P. (2019). Green Spaces and Mortality: A Systematic Review and Meta-Analysis
of Cohort Studies. Lancet Planet Health.

[18] Twohig-Bennett C., Jones A. (2018). The Health Benefits of the Great Outdoors: A Systematic Review and
Meta-Analysis of Greenspace Exposure and Health Outcomes. Environmental Research.

[19] Ulrich R. S., Simons R. F., Losito B. D., Fiorito E., Miles M. A., Zelson M. (1991). Stress Recovery during Exposure to
Natural and Urban
Environments. Journal of Environmental Psychology.

[20] Li D., Sullivan W. C. (2016). Impact
of Views to School Landscapes on Recovery from
Stress and Mental Fatigue. Landscape and Urban
Planning.

[21] Jiang B., Wang H., Larsen L., Bao F., Li Z., Pryor M. (2019). Quality of Sweatshop Factory Outdoor Environments Matters for Workers’
Stress and Anxiety: A Participatory Smartphone-Photography Survey. Journal of Environmental Psychology.

[22] Pu F., Chen W., Li C., Fu J., Gao W., Ma C., Cao X., Zhang L., Hao M., Zhou J., Huang R., Ma Y., Hu K., Liu Z. (2024). Heterogeneous
Associations of Multiplexed Environmental Factors and Multidimensional
Aging Metrics. Nat. Commun..

[23] Wang Y., Shi X., Yang F., Wang S., Han T., Ma K. (2025). Association
of Urban Green and Blue Space with Accelerated Ageing: A Cohort Study
in the UK Biobank. Ecotoxicology and Environmental
Safety.

[24] Zhao Y., Yang X., He D., Wei W., Cheng B., Zhang F. (2025). Associations between Environmental
Air Pollution, Greenspace and
Apparent Biological Aging: A Cross-Sectional Study. Geroscience.

[25] Guo C., Yang J., Ma J., Chen J., Chen S., Zheng Y., Huang B., Yu J., Li T., He S. (2024). Ambient Fine Particulate Matter and
Its Constituents May Exacerbate
the Acceleration of Aging in Adults. Environ.
Int..

[26] Pan R., Wang J., Chang W., Song J., Yi W., Zhao F., Zhang Y., Fang J., Du P., Cheng J., Li T., Su H., Shi X. (2023). Association
of PM2.5 Components with Acceleration of Aging: Moderating Role of
Sex Hormones. Environ. Sci. Technol..

[27] Liang R., Fan L., Lai X., Shi D., Wang H., Shi W., Liu W., Yu L., Song J., Wang B. (2024). Air Pollution Exposure,
Accelerated Biological Aging, and Increased Thyroid Dysfunction Risk:
Evidence from a Nationwide Prospective Study. Environ. Int..

[28] Wang H., Yang Y., Li G., Wang Y., Wu Y., Shi L., Zhu Y., Li J. (2025). Exposure to Green Space, Nighttime
Light, Air Pollution, and Noise and Cardiovascular Disease Risk: A
Prospective Cohort Study. Environ. Pollut..

[29] Wang J., Ma Y., Tang L., Li D., Xie J., Hu Y., Tian Y. (2024). Long-Term Exposure
to Residential Greenness and Decreased Risk of
Depression and Anxiety. Nat. Mental Health.

[30] Ji J. S., Zhu A., Lv Y., Shi X. (2020). Interaction between Residential Greenness
and Air Pollution Mortality: Analysis of the Chinese Longitudinal
Healthy Longevity Survey. Lancet Planetary Health.

[31] Wu Q., Li C., Zhang L., Zhou Y. (2025). The Mitigation Effects of Residential
Green Space and Low Air Pollution on Socioeconomic Inequalities in
Depression. npj Mental Health Res..

[32] Stekhoven D. J., Bühlmann P. (2012). MissForest—Non-Parametric
Missing Value Imputation
for Mixed-Type Data. Bioinformatics.

[33] de
Souza P., Anenberg S., Makarewicz C., Shirgaokar M., Duarte F., Ratti C., Durant J. L., Kinney P. L., Niemeier D. (2024). Quantifying Disparities in Air Pollution
Exposures across the United States Using Home and Work Addresses. Environ. Sci. Technol..

[34] Shekarrizfard M., Faghih-Imani A., Hatzopoulou M. (2016). An Examination of Population Exposure
to Traffic Related Air Pollution: Comparing Spatially and Temporally
Resolved Estimates against Long-Term Average Exposures at the Home
Location. Environmental Research.

[35] Gao, J. ; Shi, Y. ; Zhang, H. ; Chen, X. China Regional 250m Normalized Difference Vegetation Index Data Set (2000–2024). In National Tibetan Plateau Data Center; 2025; 10.11888/Terre.tpdc.300328.

[36] Chen B., Wu S., Song Y., Webster C., Xu B., Gong P. (2022). Contrasting
Inequality in Human Exposure to Greenspace between Cities of Global
North and Global South. Nat. Commun..

[37] Wei J., Li Z., Lyapustin A., Sun L., Peng Y., Xue W., Su T., Cribb M. (2021). Reconstructing
1-Km-Resolution High-Quality PM2.5 Data
Records from 2000 to 2018 in China: Spatiotemporal Variations and
Policy Implications. Remote Sensing of Environment.

[38] Wei J., Li Z., Xue W., Sun L., Fan T., Liu L., Su T., Cribb M. (2021). The ChinaHighPM10
Dataset: Generation, Validation,
and Spatiotemporal Variations from 2015 to 2019 across China. Environ. Int..

[39] Wei J., Li Z., Li K., Dickerson R. R., Pinker R. T., Wang J., Liu X., Sun L., Xue W., Cribb M. (2022). Full-Coverage Mapping
and Spatiotemporal Variations of Ground-Level Ozone (O3) Pollution
from 2013 to 2020 across China. Remote Sensing
of Environment.

[40] Wei J., Liu S., Li Z., Liu C., Qin K., Liu X., Pinker R. T., Dickerson R. R., Lin J., Boersma K. F., Sun L., Li R., Xue W., Cui Y., Zhang C., Wang J. (2022). Ground-Level
NO_2_ Surveillance from Space across China
for High Resolution Using Interpretable Spatiotemporally Weighted
Artificial Intelligence. Environ. Sci. Technol..

[41] Wei J., Li Z., Wang J., Li C., Gupta P., Cribb M. (2023). Ground-Level
Gaseous Pollutants (NO_2_, SO_2_, and CO) in China:
Daily Seamless Mapping and Spatiotemporal Variations. Atmospheric Chemistry and Physics.

[42] Vanoli J., Mistry M. N., De La
Cruz Libardi A., Masselot P., Schneider R., Ng C. F. S., Madaniyazi L., Gasparrini A. (2024). Reconstructing
Individual-Level Exposures in Cohort Analyses of Environmental Risks:
An Example with the UK Biobank. J. Expo Sci.
Environ. Epidemiol.

[43] Klemera P., Doubal S. (2006). A New Approach to the
Concept and Computation of Biological
Age. Mechanisms of Ageing and Development.

[44] Kwon D., Belsky D. (2021). A Toolkit for Quantification of Biological Age from
Blood Chemistry and Organ Function Test Data: BioAge. GeroScience.

[45] Chen L., Zhang Y., Yu C., Guo Y., Sun D., Pang Y., Pei P., Yang L., Millwood I. Y., Walters R. G., Chen Y., Du H., Liu Y., Burgess S., Stevens R., Chen J., Chen Z., Li L., Lv J. (2023). Modeling Biological Age Using Blood Biomarkers and
Physical Measurements in Chinese Adults. eBioMedicine.

[46] Liu Z. (2021). Development
and Validation of 2 Composite Aging Measures Using Routine Clinical
Biomarkers in the Chinese Population: Analyses from 2 Prospective
Cohort Studies. J. Gerontol A Biol. Sci. Med.
Sci..

[47] Wang K., Gao J., Liu Y., Liu Z., Li Y., Chen S., Sun L., Wu S., Gao X. (2025). Biological Age Construction for Prediction
of Mortality in the Chinese Population. GeroScience.

[48] Nagin D. S., Odgers C. L. (2010). Group-based trajectory
modeling in clinical research. Annu. Rev. Clin.
Psychol..

[49] Proust-Lima C., Philipps V., Liquet B. (2017). Estimation of Extended Mixed Models
Using Latent Classes and Latent Processes: The R Package Lcmm. Journal of Statistical Software.

[50] Galbraith S., Bowden J., Mander A. (2017). Accelerated
Longitudinal Designs:
An Overview of Modelling, Power, Costs and Handling Missing Data. Stat Methods Med. Res..

[51] Lennon H., Kelly S., Sperrin M., Buchan I., Cross A. J., Leitzmann M., Cook M. B., Renehan A. G. (2018). Framework to Construct
and Interpret Latent Class Trajectory Modelling. BMJ. Open.

[52] Mésidor M., Rousseau M.-C., O’Loughlin J., Sylvestre M.-P. (2022). Does Group-Based
Trajectory Modeling Estimate Spurious Trajectories?. BMC Medical Research Methodology.

[53] Liu X., Zhang G., Zhang G., Yang H., Ling X., Xi J., Wu W., Liu W., Zhou Z., Ren J., Cao J. (2024). Trajectory Model to
Analyze the Effect of Multi-Metal Exposures on
Sperm Parameters and Sex Hormones of the MARHCS Cohort in China. Environmental Research.

[54] Li Q.-Q., Huang J., Cai D., Chou W.-C., Zeeshan M., Chu C., Zhou Y., Lin L., Ma H.-M., Tang C., Kong M., Xie Y., Dong G.-H., Zeng X.-W. (2023). Prenatal
Exposure to Legacy and Alternative Per- and Polyfluoroalkyl Substances
and Neuropsychological Development Trajectories over the First 3 Years
of Life. Environ. Sci. Technol..

[55] Wood S. N. (2025). Generalized
Additive Models. Annual Review of Statistics
and Its Application.

[56] Carrico C., Gennings C., Wheeler D. C., Factor-Litvak P. (2015). Characterization
of Weighted Quantile Sum Regression for Highly Correlated Data in
a Risk Analysis Setting. JABES.

[57] Tingley D., Yamamoto T., Hirose K., Keele L., Imai K. (2014). Mediation: *R* Package
for Causal Mediation Analysis. J. Stat. Soft..

[58] Keil A. P., Buckley J. P., O’Brien K. M., Ferguson K. K., Zhao S., White A. J. (2020). A Quantile-Based g-Computation Approach to Addressing
the Effects of Exposure Mixtures. Environ. Health
Perspect.

[59] VanderWeele T. J., Ding P. (2017). Sensitivity
Analysis in Observational Research: Introducing the E-Value. Ann. Int. Med..

[60] Ryan J., Wrigglesworth J., Loong J., Fransquet P. D., Woods R. L. (2020). A Systematic Review
and Meta-Analysis of Environmental,
Lifestyle, and Health Factors Associated with DNA Methylation Age. J. Gerontol A Biol. Sci. Med. Sci..

[61] Markevych I., Schoierer J., Hartig T., Chudnovsky A., Hystad P., Dzhambov A. M., de Vries S., Triguero-Mas M., Brauer M., Nieuwenhuijsen M. J., Lupp G., Richardson E. A., Astell-Burt T., Dimitrova D., Feng X., Sadeh M., Standl M., Heinrich J., Fuertes E. (2017). Exploring Pathways
Linking Greenspace to Health: Theoretical and Methodological Guidance. Environmental Research.

[62] Tiwari A., Kumar P., Baldauf R., Zhang K. M., Pilla F., Di Sabatino S., Brattich E., Pulvirenti B. (2019). Considerations
for Evaluating Green Infrastructure Impacts in Microscale and Macroscale
Air Pollution Dispersion Models. Science of
The Total Environment.

[63] Bell M. L., Zanobetti A., Dominici F. (2014). Who Is More Affected by Ozone Pollution?
A Systematic Review and Meta-Analysis. Am. J.
Epidemiol.

[64] Fuentes N., Nicoleau M., Cabello N., Montes D., Zomorodi N., Chroneos Z. C., Silveyra P. (2019). 17β-Estradiol Affects Lung
Function and Inflammation Following Ozone Exposure in a Sex-Specific
Manner. American Journal of Physiology. Lung
Cellular and Molecular Physiology.

[65] Ward
Thompson C., Roe J., Aspinall P., Mitchell R., Clow A., Miller D. (2012). More Green Space Is Linked to Less
Stress in Deprived Communities: Evidence from Salivary Cortisol Patterns. Landscape and Urban Planning.

[66] Kuh D., Ben-Shlomo Y., Lynch J., Hallqvist J., Power C. (2003). Life Course Epidemiology. Journal of Epidemiology
and Community Health.

