# Supplementary material for: Associations of Green Space and Air Pollution Mixture with Heterogeneous Biological Aging Patterns
Source: Environ Health Perspect. 2026 Sep 3;134(6):659–71. doi: 10.1021/EHP.6c00004 (PMC13648064; doi:10.1021/EHP.6c00004)
Supplement: Supplementary file 1 [file hp6c00004_si_001.pdf]

Supplementary Information

Associations of Green Space and Air Pollution Mixture with Heterogeneous Biological Aging Patterns

Guoqiang Ma[a], Jing Xu[a], Yishu Li[a], Yuefei Wu[a], Qian Li[a], Xuan Cao[a], Zitong Zhao[a], Jingyan Gao[a], Lijun Gao[b], Lina Yan[a]*, Xiaolin Zhang[a]*

[a]Department of Epidemiology and Statistics, School of Public Health, Hebei Medical University, Hebei Province Key Laboratory of Environment and Human Health, Shijiazhuang 050017, China
[b]Department of Occupational Medicine, the Eighth Hospital of Hebei Province, Shijiazhuang 050021, China

*Corresponding author: Xiaolin Zhang, E-mail: 17700862@hebmu.edu.cn. Tel: 0311-86265588; Lina Yan, E-mail: 503622294@qq.com, Tel: 0311-86265588

Contents

Supplementary Methods 5

S1.1 Calculation of Klemera-Doubal Method (KDM) Biological Age 5

S1.2. Group-Based Trajectory Modeling (GBTM) for Aging Trajectories 6

Supplementary Tables 8

Table S1. Pearson's correlation coefficients between CA and blood biomarkers or physical measurements stratified by sex. 8

Table S2. Model fit statistics for GBTM with different numbers of latent trajectory groups. 9

Table S3. Nested models for the interaction between green space (NDVI) and the air pollution mixture (WQS index). 10

Table S4. Sensitivity analyses for the air pollution mixture effect using alternative models and parameter specifications. 11

Table S5. Sex-stratified associations of green space exposures with aging trajectories. 12

Table S6. Sex-stratified associations of air pollution exposures with aging trajectories. 13

Table S7. Age-stratified associations of green space exposures with aging trajectories. 14

Table S8. Age-stratified associations of air pollution exposures with aging trajectories. 15

Table S9. Associations of quantiled green space exposures with aging trajectories. ... 16

Table S10. Associations of quantiled air pollutant exposures with aging trajectories. 17

Table S11. Sensitivity analysis for the associations of green space exposures with aging trajectories, restricted to the complete-case dataset. 18

Table S12. Sensitivity analysis for the associations of air pollution exposures with aging trajectories, restricted to the complete-case dataset. 19

Table S13. Cross-classification of trajectory groups by GBTM and GMM. 20

Table S14. E-value for Unmeasured Confounding in Main Associations. 21

Table S15. Quantitative comparison of expected linear and non-linear odds-ratio estimates (Odds Ratios) at specific exposure percentiles relative to the median. 22

Supplementary Figures 24

Figure S1. Flow diagram of participant selection. 26

Figure S2. Distribution and correlation of chronological and biological ages. 27

Figure S3. Model fit and classification quality indices for group-based trajectory models (GBTM) with different numbers of latent trajectory groups. 28

Figure S4. Temporal trends of annual average air pollutant concentrations in the study area (2020–2023). 29

Figure S5. Spearman's correlation coefficients among long-term air pollutants (calculated at the workplace level). 30

Figure S6. High-resolution spatial distribution of 3-year mean environmental exposures across the study area. 31

Figure S7. Associations between long-term environmental exposures and continuous biological age acceleration (BAA) derived from linear mixed models. 32

Figure S8. Sensitivity analysis using a 1-year lagged exposure window (2020–2022) for green space exposures. 33

Figure S9. Sensitivity analysis using a 1-year lagged exposure window (2020–2022) for air pollution exposures. 34

Figure S10. Sensitivity analysis for the associations between the Enhanced Vegetation Index (EVI) at multiple buffer sizes and biological aging trajectories. 35

Figure S11. Pollutant weights from the Weighted Quantile Sum (WQS) regression model with 10 quantiles. 36

Figure S12. Pollutant weights from the Quantile G-Computation (QGC) model. 37

Figure S13. Associations of green space and air pollutants with aging trajectories after additional adjustment for occupational categories. 38

Figure S14. Comparison of generalized additive mixed models (GAMM) and generalized linear mixed models (GLMM) with rug plots. 39

Supplementary Methods

S1.1 Calculation of Klemera-Doubal Method (KDM) Biological Age

We calculated a sex-specific Klemera-Doubal Method (KDM) Biological Age (BA) for each participant at each visit. The KDM algorithm is derived from a series of linear regressions of individual biomarkers on chronological age (CA) in a reference population. It integrates information from multiple physiological systems to estimate systemic aging [1]. The general formula is as follows:

$$\mathrm{KDMAge} = \frac{\sum_{i=1}^{m}(x_i - q_i)\left(\frac{k_i}{s_i^2}\right) + \frac{CA}{s_{BA}^2}}{\sum_{i=1}^{m}\left(\frac{k_i}{s_i^2}\right) + \frac{1}{s_{BA}^2}}$$

In this equation:

- x_i is the measurement of biomarker i for a given participant.
- The parameters k_i, q_i, and s_i are the slope, intercept, and root mean squared error (RMSE), respectively. These were estimated from separate linear regression models of CA on each biomarker i (CA ~ biomarker_i) within the sex-specific reference population (NHANES 2015–2018).
- s_BA is a scaling factor, defined as the square root of the variance in CA that is explained by the full set of selected biomarkers in the reference population.

The final set of 15 biomarkers used in our models was selected based on a two-step protocol: (1) significant correlation with CA ($|r| > 0.1$), and (2) To address high inter-correlations among the selected biomarkers, we implemented an "either-or" selection strategy. For any pair of biomarkers with a correlation coefficient $|r| \geq 0.7$, we retained only the one that had a stronger correlation with CA, and excluded the other[2]. A complete list of the included biomarkers is provided in Supplementary Table S1. The BioAge package in R was used to facilitate the calculation.

S1.2. Group-Based Trajectory Modeling (GBTM) for Aging Trajectories

We used group-based trajectory modeling (GBTM) to identify latent classes of individuals with similar longitudinal patterns of Biological Age Acceleration (BAA)[3]. We fitted a series of models with the number of trajectory groups (ng) ranging from one to five, implemented in the lcmm R package[4]. Each model specified BAA as a quadratic function of age to capture potential non-linear patterns.

The optimal number of groups was determined by a comprehensive assessment of multiple model fit and classification quality criteria[5]. These included:

- Information Criteria: The Akaike Information Criterion (AIC) and the Bayesian Information Criterion (BIC), where lower values indicate a better model fit.
- Classification Certainty: The average posterior probability of assignment (APPA) for each group, with values > 0.70 considered acceptable, and the overall Integrated Completed Likelihood (ICL) criterion, which penalizes models with poor classification (lower ICL is better).
- Group Size and Interpretability: The proportion of individuals assigned to each group (with each group required to contain at least 5% of the total sample), and the clinical interpretability and distinctiveness of the trajectory shapes.

As shown in Supplementary Table S2, the three-group model provided the optimal balance across all criteria. It offered a substantial reduction in AIC and BIC compared to the two-group model, while the four-group model showed only marginal improvement and had poorer classification quality (lower APPA and higher ICL). Furthermore, the three-group solution yielded trajectories that were clearly distinct and clinically meaningful, representing “Slow”, “Moderate”, and “Accelerated” aging patterns (Figure 1 and Supplementary Figure S2).

References

[1] Kwon D, Belsky D. A toolkit for quantification of biological age from blood chemistry and organ function test data: BioAge. GeroScience 2021;43. https://doi.org/10.1007/s11357-021-00480-5.

[2] Liu Z. Development and validation of 2 composite aging measures using routine clinical biomarkers in the Chinese population: Analyses from 2 prospective cohort studies n.d.

[3] Nagin DS, Odgers CL. Group-based trajectory modeling in clinical research. Annu Rev Clin Psychol 2010;6:109–38. https://doi.org/10.1146/annurev.clinpsy.121208.131413.

[4] Proust-Lima C, Philipps V, Liquet B. Estimation of extended mixed models using latent classes and latent processes: The R package lcmm. Journal of Statistical Software 2017;78:1–56. https://doi.org/10.18637/jss.v078.i02.

[5] Nylund KL, Asparouhov T, Muthén BO. Deciding on the number of classes in latent class analysis and growth mixture modeling: A monte carlo simulation study. Structural Equation Modeling: A Multidisciplinary Journal 2007;14:535–69. https://doi.org/10.1080/10705510701575396.

Supplementary Tables

Table S1. Pearson's correlation coefficients between CA and blood biomarkers or physical measurements stratified by sex.

	Male		Female	
	r	***P***	***r***	***P***
Biomarkers				
Albumin, g/L	**-0.465**	<0.001	**-0.171**	<0.001
Blood Urea Nitrogen，mg/dL	**0.178**	<0.001	**0.317**	<0.001
Red Cell Distribution Width，%	**0.248**	<0.001	-0.050	<0.001
Diastolic blood pressure, mmHg	**0.111**	<0.001	**0.256**	<0.001
High density lipoprotein cholesterol, mg/dL	0.042	<0.001	-0.029	0.007
Low density lipoprotein cholesterol, mg/dL	-0.043	<0.001	**0.343**	<0.001
Lymphocyte percent，%	**-0.129**	<0.001	0.001	0.912
Mean cell volume, fL	**0.293**	<0.001	**0.172**	<0.001
Red blood cell count，million cells/uL	**-0.207**	<0.001	0.015	0.153
Systolic blood pressure, mmHg	**0.363**	<0.001	**0.528**	<0.001
Total cholesterol, mg/dL	-0.038	0.001	**0.332**	<0.001
Total triglyceride, mg/dL	-0.047	<0.001	**0.334**	<0.001
Uric acid, mg/dL	**-0.206**	<0.001	**0.240**	<0.001
HbA1c, %	**0.273**	<0.001	**0.414**	<0.001
Creatinine, mg/dL	0.071	<0.001	**0.239**	<0.001
Body mass index, kg/m2	-0.036	0.002	**0.258**	<0.001
Gamma glutamyl transferase, U/L	**0.261**	<0.001	**0.350**	<0.001
Cystatin C, mg/L	**0.351**	<0.001	**0.382**	<0.001
Basophils percent, %	-0.001	0.829	-0.005	0.618
Eosinophils percent, %	-0.009	0.482	0.003	0.151
Total bilirubin, mg/dL	0.032	0.017	0.058	<0.001

Data are Pearson's correlation coeffici *P*-values from the baseline visit of the study population (N=11,760), stratified by sex. This table illustrates the first step of our two-stage model-building process. Biomarkers with $|r| > 0.1$ were initially selected. For pairs with $|r| > 0.7$, the one with a stronger correlation to CA was retained. The final 15 biomarkers selected through this process (shown in bold) were then used to estimate model parameters in the external NHANES reference population.

Table S2. Model fit statistics for GBTM with different numbers of latent trajectory groups.

G	loglik	conv	npm	AIC	BIC	SABIC	Entropy	ICL1	ICL2	class1 (%)	class2 (%)	class3 (%)	class4 (%)	class5 (%)
1	-143,710.0	1	4	287,428.1	287,458.9	287,446.2	1.0000000	287,458.9	287,458.9	100.00000				
2	-138,081.0	1	8	276,178.1	276,239.7	276,214.3	0.7149750	279,480.8	279,562.0	60.45718	39.542822			
**3**	-136,356.4	1	12	272,736.8	**272,829.3**	272,791.1	**0.7031208**	**278,179.8**	**278,343.4**	31.80128	49.466626	18.73209		
4	-136,036.5	1	16	272,104.9	272,228.2	272,177.4	0.6301765	280,638.8	281,121.6	20.06096	39.451387	28.08290	12.40475	
5	-136,017.7	1	20	272,075.4	272,229.5	272,166.0	0.5694964	283,596.0	284,557.1	19.32338	1.103322	26.30905	41.40811	11.85614

Abbreviation: AIC, Akaike Information Criterion; BIC, Bayesian Information Criterion; SABIC, Sample-Adjusted Bayesian Information Criterion; ICL, Integrated Completed Likelihood Criterion.

The three-group model (in bold) was selected based on the optimal balance of BIC, ICL, entropy, and group size.

Table S3. Nested models for the interaction between green space (NDVI) and the air pollution mixture (WQS index).

	Model 1: WQS Only			Model 2: Main Effects			Model 3: Interaction		
**Variable**	**OR**	**95% CI**	***P* value**	**OR**	**95% CI**	***P* value**	**OR**	**95% CI**	***P* value**
**WQS Index (per SD)**	1.16	1.08-1.24	**<0.001**	1.14	1.06-1.23	**<0.001**	1.15	1.07-1.24	**<0.001**
**NDVI (per SD)**				0.88	0.79-0.98	**0.019**	0.88	0.78-0.99	**0.012**
**WQS Index × NDVI Interaction**							1.02	0.92-1.13	0.700
AIC	10,347			10,343			10,345		
Adjusted R-squared	0.062			0.062			0.062		

Abbreviations: OR, Odds Ratio, CI, Confidence Interval

All models were adjusted for sex, baseline age, smoking status, alcohol consumption, prevalent chronic diseases and individual-level socioeconomic status at baseline. All models included a random intercept for workplace to account for data clustering.

Table S4. Sensitivity analyses for the air pollution mixture effect using alternative models and parameter specifications.

Analysis Type	Outcome Comparison	Quantiles (q)	Model	OR (95% CI)	*P*
**Primary Analysis**	Accelerated vs. non-Accelerated	4	Binary WQS	1.16 (1.08, 1.24)	<0.001
**Sensitivity Analyses**					
Alternative Parameter	Accelerated vs. non-Accelerated	10	Binary WQS	1.10 (1.04, 1.17)	<0.001
Alternative Model	Accelerated vs. non-Accelerated	4	Binary QGC	1.30 (1.16, 1.47)	<0.001
	Moderate vs. Slow Aging	4	Multinomial QGC	1.14 (1.03, 1.26)	0.014
	Accelerated vs. Slow Aging	4	Multinomial QGC	1.39 (1.20, 1.60)	<0.001

Abbreviation: CI, confidence interval; OR, odds ratio; WQS, Weighted Quantile Sum regression; QGC, Quantile G- Computation.

All models were adjusted for sex, baseline age, smoking status, alcohol consumption, prevalent chronic diseases and individual-level socioeconomic status at baseline. All models included a random intercept for workplace to account for data clustering. ORs and 95% CIs were derived from the mixture slope parameter (psi) for each model.

The Primary Analysis row shows the main result from the binary WQS model using 4 quantiles for comparison.

Table S5. Sex-stratified associations of green space exposures with aging trajectories.

Green space	Male		Female		
	OR (95% CI)	*P* value	OR (95% CI)	*P* value	*P* for interaction
**Moderate vs. Slow**					
NDVI 250m	1.05 (0.98, 1.13)	0.161	0.94 (0.86, 1.02)	0.159	0.072
NDVI 500m	1.06 (0.98, 1.14)	0.138	0.94 (0.86, 1.03)	0.173	0.069
NDVI 750m	1.07 (0.99, 1.15)	0.090	0.94 (0.86, 1.03)	0.164	0.028
NDVI 1000m	1.07 (0.99, 1.16)	0.071	0.93 (0.85, 1.02)	0.136	0.021
**Accelerated vs. Slow**					
NDVI 250m	0.89 (0.78, 1.02)	0.097	0.88 (0.77, 1.02)	0.085	0.955
NDVI 500m	0.89 (0.77, 1.02)	0.094	0.86 (0.74, 0.99)	0.047	0.671
NDVI 750m	0.91 (0.80, 1.05)	0.207	0.86 (0.75, 1.00)	0.053	0.426
NDVI 1000m	0.94 (0.82, 1.08)	0.391	0.87 (0.75, 1.01)	0.063	0.285

Abbreviation: OR, odds ratio; CI, confidence interval.

All models were adjusted for baseline age, smoking status, alcohol consumption, prevalent chronic diseases and individual-level socioeconomic status at baseline, excluding sex which was the stratification variable. All models included a random intercept for workplace to account for data clustering. Exposures were standardized (per 1-SD increase). ORs and 95% CIs were derived from multinomial logistic mixed-effects models. *P* for interaction was derived from the Wald test for the product term between the exposure and sex.

Table S6. Sex-stratified associations of air pollution exposures with aging trajectories.

Air pollution	Male		Female		
	OR (95% CI)	*P* value	OR (95% CI)	*P* value	*P* for interaction
**Moderate vs. Slow**					
$PM_{2.5}$	1.01 (0.94, 1.07)	0.873	1.07 (1.00, 1.14)	0.049	0.287
PM_{10}	1.10 (1.03, 1.18)	0.003	1.04 (0.99, 1.10)	0.143	0.164
NO_2	1.06 (0.99, 1.13)	0.055	1.06 (0.99, 1.13)	0.102	0.932
SO_2	1.00 (0.94, 1.07)	0.986	1.08 (1.00, 1.16)	0.044	0.255
O_3	0.94 (0.87, 1.01)	0.075	1.10 (1.01, 1.19)	0.032	0.017
CO	1.01 (0.94, 1.09)	0.750	1.05 (0.95, 1.15)	0.342	0.603
**Accelerated vs. Slow**					
$PM_{2.5}$	1.04 (0.94, 1.15)	0.472	1.16 (1.06, 1.27)	<0.001	0.147
PM_{10}	1.21 (1.10, 1.34)	<0.001	1.09 (1.01, 1.17)	0.024	0.056
NO_2	0.97 (0.87, 1.08)	0.601	1.13 (1.02, 1.24)	0.020	0.734
SO_2	1.11 (0.99, 1.25)	0.078	1.16 (1.03, 1.29)	0.012	0.304
O_3	1.00 (0.88, 1.14)	0.947	1.25 (1.10, 1.42)	<0.001	0.003
CO	1.01 (0.88, 1.16)	0.884	1.08 (0.92, 1.26)	0.359	0.609

Abbreviation: OR, odds ratio; CI, confidence interval.

All models were adjusted for baseline age, smoking status, alcohol consumption, prevalent chronic diseases and individual-level socioeconomic status at baseline, excluding sex which was the stratification variable. All models included a random intercept for workplace to account for data clustering. Exposures were standardized (per 1-SD increase). ORs and 95% CIs were derived from multinomial logistic mixed-effects models. *P* for interaction was derived from the Wald test for the product term between the exposure and sex.

Table S7. Age-stratified associations of green space exposures with aging trajectories.

Green space	Age < 40 years		Age ≥ 40 years		
	OR (95% CI)	*P* value	OR (95% CI)	*P* value	*P* for interaction
**Moderate vs. Slow**					
NDVI 250m	1.05 (0.94, 1.16)	0.399	0.98 (0.94, 1.05)	0.995	0.825
NDVI 500m	1.06 (0.95, 1.19)	0.292	0.98 (0.94, 1.04)	0.845	0.796
NDVI 750m	1.06 (0.94, 1.19)	0.270	0.99 (0.94, 1.06)	0.861	0.655
NDVI 1000m	1.06 (0.95, 1.18)	0.291	0.99 (0.94, 1.05)	0.805	0.568
**Accelerated vs. Slow**					
NDVI 250m	0.87 (0.73, 1.03)	0.111	0.90 (0.81, 1.02)	0.110	0.004
NDVI 500m	0.86 (0.71, 1.03)	0.105	0.89 (0.79, 0.99)	0.049	0.016
NDVI 750m	0.87 (0.73, 1.05)	0.156	0.90 (0.80, 1.01)	0.075	0.026
NDVI 1000m	0.89 (0.74, 1.07)	0.212	0.91 (0.80, 1.02)	0.116	0.031

Abbreviation: OR, odds ratio; CI, confidence interval.

All models were adjusted for sex, baseline age, smoking status, alcohol consumption, prevalent chronic diseases and individual-level socioeconomic status at baseline. All models included a random intercept for workplace to account for data clustering. Age was the stratification variable. Exposures were standardized (per 1-SD increase). ORs and 95% CIs were derived from multinomial logistic mixed-effects models. *P* for interaction was derived from the Wald test for the product term between the exposure and age group.

Table S8. Age-stratified associations of air pollution exposures with aging trajectories.

Air pollution	Age < 40 years		Age ≥ 40 years		
	OR (95% CI)	*P* value	OR (95% CI)	*P* value	*P* for interaction
**Moderate vs Slow**					
$PM_{2.5}$	1.04 (0.96, 1.12)	0.873	1.03 (0.97, 1.09)	0.240	0.697
PM_{10}	1.10 (1.04, 1.17)	0.002	1.04 (0.98, 1.10)	0.250	0.198
NO_2	1.08 (1.00, 1.17)	0.040	1.01 (0.96, 1.07)	0.639	0.023
SO_2	0.99 (0.91, 1.08)	0.860	1.05 (1.00, 1.11)	0.047	0.407
O_3	0.96 (0.87, 1.08)	0.567	1.03 (0.98, 1.10)	0.179	0.664
CO	0.99 (0.88, 1.11)	0.819	1.00 (0.95, 1.06)	0.946	0.364
**Accelerated vs Slow**					
$PM_{2.5}$	1.04 (0.96, 1.12)	0.313	1.11 (1.01, 1.21)	0.020	0.137
PM_{10}	1.21 (1.10, 1.31)	<0.001	1.09 (1.00, 1.18)	0.054	0.108
NO_2	1.13 (1.00, 1.27)	0.037	0.97 (0.88, 1.07)	0.585	0.006
SO_2	1.07 (0.93, 1.23)	0.360	1.15 (1.04, 1.26)	0.005	0.547
O_3	1.03 (0.86, 1.23)	0.739	1.18 (1.06, 1.32)	0.002	0.404
CO	1.05 (0.88, 1.25)	0.598	0.99 (0.87, 1.12)	0.858	0.007

Abbreviation: OR, odds ratio; CI, confidence interval.

All models were adjusted for sex, baseline age, smoking status, alcohol consumption, prevalent chronic diseases and individual-level socioeconomic status at baseline. All models included a random intercept for workplace to account for data clustering. Age was the stratification variable. Exposures were standardized (per 1-SD increase). ORs and 95% CIs were derived from multinomial logistic mixed-effects models. *P* for interaction was derived from the Wald test for the product term between the exposure and age group.

Table S9. Associations of quantiled green space exposures with aging trajectories.

	Q1	Q2		Q3		Q4	
		OR (95% CI)	*P* value	OR (95% CI)	*P* value	OR (95% CI)	*P* value
**Moderate vs Slow Aging**							
NDVI 250m	1 (ref.)	0.96 (0.75, 1.23)	0.740	1.04 (0.86, 1.25)	0.697	0.98 (0.81, 1.18)	0.852
NDVI 500m	1 (ref.)	1.30 (0.96, 1.70)	0.059	1.02 (0.86, 1.22)	0.785	0.96 (0.81, 1.14)	0.646
NDVI 750m	1 (ref.)	0.78 (0.28, 2.17)	0.639	0.93 (0.79, 1.09)	0.373	0.90 (0.77, 1.05)	0.194
NDVI 1000m	1 (ref.)	1.08 (0.79, 1.47)	0.624	1.10 (0.93, 1.31)	0.271	0.97 (0.83, 1.14)	0.711
**Accelerated vs Slow Aging**							
NDVI 250m	1 (ref.)	1.01 (0.64, 1.59)	0.959	1.11 (0.78, 1.57)	0.562	0.80 (0.56, 1.13)	0.208
NDVI 500m	1 (ref.)	1.30 (0.81, 2.07)	0.275	1.25 (0.90, 1.74)	0.178	0.79 (0.58, 1.08)	0.140
NDVI 750m	1 (ref.)	2.55 (0.83, 7.85)	0.102	1.11 (0.82, 1.50)	0.516	0.74 (0.56, 0.99)	0.039
NDVI 1000m	1 (ref.)	0.80 (0.32, 1.99)	0.637	1.16 (0.85, 1.58)	0.337	0.75 (0.57, 1.00)	0.052

Abbreviation: OR, odds ratio; CI, confidence interval; Q, quartile.

All models were adjusted for sex, baseline age, smoking status, alcohol consumption, prevalent chronic diseases and individual-level socioeconomic status at baseline. All models included a random intercept for workplace to account for data clustering. ORs and 95% CIs compare each higher quartile (Q2, Q3, Q4) of exposure to the lowest quartile (Q1).

Table S10. Associations of quantiled air pollutant exposures with aging trajectories.

	Q1	Q2		Q3		Q4	
		OR (95% CI)	*P* value	OR (95% CI)	*P* value	OR (95% CI)	*P* value
**Moderate vs Slow**							
$PM_{2.5}$	1 (ref.)	1.01 (0.89, 1.16)	0.846	1.12 (0.95, 1.31)	0.165	1.16 (0.99, 1.34)	0.058
PM_{10}	1 (ref.)	0.92 (0.79, 1.07)	0.276	0.98 (0.87, 1.11)	0.801	1.14 (1.00, 1.31)	0.051
SO_2	1 (ref.)	0.98 (0.84, 1.15)	0.844	0.97 (0.73, 1.28)	0.811	1.03 (0.89, 1.19)	0.711
NO_2	1 (ref.)	0.99 (0.84, 1.16)	0.903	1.12 (0.97, 1.29)	0.121	1.17 (1.01, 1.35)	0.040
O_3	1 (ref.)	1.01 (0.88, 1.17)	0.850	1.06 (0.78, 1.45)	0.696	0.97 (0.82, 1.16)	0.763
CO	1 (ref.)	0.98 (0.85, 1.12)	0.734	1.10 (0.92, 1.30)	0.289	1.17 (0.81, 1.70)	0.407
**Accelerated vs Slow**							
$PM_{2.5}$	1 (ref.)	0.94 (0.75, 1.17)	0.563	1.19 (0.94, 1.50)	0.154	1.30 (1.03, 1.64)	0.025
PM_{10}	1 (ref.)	0.87 (0.70, 1.08)	0.202	0.89 (0.74, 1.06)	0.180	1.31 (1.08, 1.59)	0.005
SO_2	1 (ref.)	1.27 (0.97, 1.66)	0.081	1.12 (0.67, 1.86)	0.674	1.44 (1.11, 1.88)	0.006
NO_2	1 (ref.)	0.75 (0.56, 1.01)	0.053	1.02 (0.79, 1.32)	0.899	1.03 (0.78, 1.35)	0.835
O_3	1 (ref.)	1.13 (0.87, 1.48)	0.353	0.95 (0.38, 2.38)	0.919	1.26 (0.90, 1.75)	0.174
CO	1 (ref.)	0.89 (0.68, 1.16)	0.391	1.06 (0.74, 1.51)	0.765	0.96 (0.52, 1.76)	0.887

Abbreviation: OR, odds ratio; CI, confidence interval; Q, quartile.

All models were adjusted for sex, baseline age, smoking status, alcohol consumption, prevalent chronic diseases and individual-level socioeconomic status at baseline. All models included a random intercept for workplace to account for data clustering. ORs and 95% CIs compare each higher quartile (Q2, Q3, Q4) of exposure to the lowest quartile (Q1).

Table S11. Sensitivity analysis for the associations of green space exposures with aging trajectories, restricted to the complete-case dataset.

**Greenspace**	**Comparison**	**OR (95% CI)**	***P* value**
NDVI 250m	Moderate vs. Slow	0.991 (0.935, 1.051)	0.763
	Accelerated vs. Slow	0.887 (0.787, 0.998)	0.047
NDVI 500m	Moderate vs. Slow	0.992 (0.934, 1.054)	0.803
	Accelerated vs. Slow	0.869 (0.768, 0.984)	0.026
NDVI 750m	Moderate vs. Slow	0.996 (0.938, 1.056)	0.883
	Accelerated vs. Slow	0.882 (0.779, 0.999)	0.048
NDVI 1000m	Moderate vs. Slow	0.996 (0.937, 1.057)	0.887
	Accelerated vs. Slow	0.895 (0.788, 1.017)	0.089

Abbreviation: OR, odds ratio; CI, confidence interval.

This analysis was restricted to participants with no missing data on any of the included analytical variables (N=9500). All models were adjusted for sex, baseline age, smoking status, alcohol consumption, prevalent chronic diseases and individual-level socioeconomic status. All models included a random intercept for workplace to account for data clustering. Exposures were standardized (per 1-SD increase). ORs and 95% CIs were derived from multinomial logistic mixed-effects models.

Table S12. Sensitivity analysis for the associations of air pollution exposures with aging trajectories, restricted to the complete-case dataset.

Pollutant	Comparison	OR (95% CI)	*P* value
$PM_{2.5}$	Moderate vs. Slow	1.04 (0.98, 1.09)	0.182
	Accelerated vs. Slow	1.11 (1.03, 1.19)	0.009
PM_{10}	Moderate vs. Slow	1.08 (1.03, 1.13)	0.003
	Accelerated vs. Slow	1.14 (1.06, 1.22)	<0.001
NO_2	Moderate vs. Slow	1.07 (1.02, 1.12)	0.011
	Accelerated vs. Slow	1.05 (0.96, 1.14)	0.288
SO_2	Moderate vs. Slow	1.02 (0.97, 1.07)	0.489
	Accelerated vs. Slow	1.13 (1.05, 1.23)	0.002
O_3	Moderate vs. Slow	1.01 (0.95, 1.07)	0.751
	Accelerated vs. Slow	1.13 (1.01, 1.26)	0.038
CO	Moderate vs. Slow	1.02 (0.96, 1.09)	0.497
	Accelerated vs. Slow	1.02 (0.89, 1.16)	0.751

Abbreviation: OR, odds ratio; CI, confidence interval.

This analysis was restricted to participants with no missing data on any of the included analytical variables (N=9500). All models were adjusted for sex, baseline age, smoking status, alcohol consumption, prevalent chronic diseases and individual-level socioeconomic status. All models included a random intercept for workplace to account for data clustering. Exposures were standardized (per 1-SD increase). ORs and 95% CIs were derived from multinomial logistic mixed-effects models.

Table S13. Cross-classification of trajectory groups by GBTM and GMM.

GBTM/GMM	Slow Aging	Moderate Aging	Accelerated Aging
Slow Aging, n (%)	5217(100)	0(0)	0(0)
Moderate Aging, n (%)	250(3.08)	7865(96.92)	0(0)
Accelerated Aging, n (%)	0(0)	263(8.56)	2810(91.44)
Total	5467	265	2810
unweighted Cohen's Kappa: 0.95 (95% CI: 0.94-0.95)			

Abbreviations: GBTM, Group-Based Trajectory Modeling; GMM, Growth Mixture Model.

The table shows the number of individuals (row percentage) in each cell. The overall agreement was 95.8%. The unweighted Cohen's Kappa coefficient was 0.95 (95% CI: 0.94-0.95), and the weighted Cohen's Kappa was 0.97 (95% CI: 0.96-0.97), both indicating almost perfect agreement between the two modeling approaches.

Table S14. E-value for Unmeasured Confounding in Main Associations.

Exposure	Contrast	OR (95%CI)	E value
$PM_{2.5}$	Slow vs. Moderate Aging	1.04(0.99,1.10)	-
	Slow vs. Accelerated Aging	1.13(1.05,1.21)	1.32
PM_{10}	Slow vs. Moderate Aging	1.06(1.02,1.11)	1.20
	Slow vs. Accelerated Aging	1.13(1.06,1.20)	1.32
NO_2	Slow vs. Moderate Aging	1.05(1.00,1.10)	1.18
	Slow vs. Accelerated Aging	1.06(0.98,1.15)	-
SO_2	Slow vs. Moderate Aging	1.04(0.99,1.10)	-
	Slow vs. Accelerated Aging	1.15(1.05,1.26)	1.34
O_3	Slow vs. Moderate Aging	1.02(0.96, 1.08)	-
	Slow vs. Accelerated Aging	1.15(1.03,1.28)	1.35
CO	Slow vs. Moderate Aging	1.03(0.96,1.10)	-
	Slow vs. Accelerated Aging	1.02(0.91,1.16)	-
NDVI 250	Slow vs. Moderate Aging	0.99(0.94,1.06)	-
	Slow vs. Accelerated Aging	0.87(0.77,0.97)	1.35
NDVI 500	Slow vs. Moderate Aging	0.99(0.93,1.06)	-
	Slow vs. Accelerated Aging	0.84(0.75,0.95)	1.39
NDVI 750	Slow vs. Moderate Aging	0.99(0.94,1.06)	-
	Slow vs. Accelerated Aging	0.86(0.76,0.97)	1.37
NDVI 1000	Slow vs. Moderate Aging	0.99(0.94,1.06)	-
	Slow vs. Accelerated Aging	0.87(0.77,0.98)	1.35

Abbreviation: OR, odds ratio; CI, confidence interval.

The E-value indicates the minimum strength of association an unmeasured confounder would need to have with both exposure and outcome to fully explain away the observed association. E-values were calculated only for statistically significant associations.

Table S15. Comparison of GLMM- and GAMM-derived odds ratios at selected exposure percentiles relative to the median.

Pollutant	Comparison	Percentile	GLMM	GAMM
$PM_{2.5}$	Moderate vs. Slow	P10	0.94 (0.77, 1.13)	0.95 (0.78, 1.15)
$PM_{2.5}$	Moderate vs. Slow	P25	0.97 (0.81, 1.16)	0.97 (0.80, 1.16)
$PM_{2.5}$	Moderate vs. Slow	P75	1.04 (0.86, 1.25)	1.05 (0.87, 1.28)
$PM_{2.5}$	Moderate vs. Slow	P90	1.04 (0.86, 1.25)	1.05 (0.87, 1.28)
$PM_{2.5}$	Accelerated vs. Slow	P10	0.83 (0.40, 1.76)	0.88 (0.41, 1.88)
$PM_{2.5}$	Accelerated vs. Slow	P25	0.92 (0.43, 1.93)	1.02 (0.47, 2.20)
$PM_{2.5}$	Accelerated vs. Slow	P75	1.10 (0.52, 2.35)	1.26 (0.58, 2.75)
$PM_{2.5}$	Accelerated vs. Slow	P90	1.11 (0.52, 2.36)	1.27 (0.58, 2.78)
PM_{10}	Moderate vs. Slow	P10	0.98 (0.82, 1.18)	1.03 (0.85, 1.25)
PM_{10}	Moderate vs. Slow	P25	1.00 (0.83, 1.20)	1.00 (0.83, 1.21)
PM_{10}	Moderate vs. Slow	P75	1.13 (0.94, 1.37)	1.15 (0.95, 1.40)
PM_{10}	Moderate vs. Slow	P90	1.14 (0.94, 1.37)	1.16 (0.95, 1.40)
PM_{10}	Accelerated vs. Slow	P10	0.96 (0.46, 2.02)	1.12 (0.53, 2.38)
PM_{10}	Accelerated vs. Slow	P25	1.00 (0.48, 2.08)	1.01 (0.48, 2.13)
PM_{10}	Accelerated vs. Slow	P75	1.27 (0.60, 2.68)	1.33 (0.63, 2.83)
PM_{10}	Accelerated vs. Slow	P90	1.28 (0.61, 2.70)	1.35 (0.64, 2.87)
NO_2	Moderate vs. Slow	P10	0.93 (0.77, 1.13)	0.96 (0.79, 1.17)
NO_2	Moderate vs. Slow	P25	0.99 (0.83, 1.18)	0.98 (0.82, 1.19)
NO_2	Moderate vs. Slow	P75	1.07 (0.89, 1.28)	1.10 (0.91, 1.33)
NO_2	Moderate vs. Slow	P90	1.07 (0.89, 1.29)	1.10 (0.91, 1.33)
NO_2	Accelerated vs. Slow	P10	0.92 (0.44, 1.94)	1.06 (0.50, 2.26)
NO_2	Accelerated vs. Slow	P25	0.98 (0.47, 2.06)	0.98 (0.46, 2.11)
NO_2	Accelerated vs. Slow	P75	1.08 (0.51, 2.29)	1.19 (0.55, 2.57)
NO_2	Accelerated vs. Slow	P90	1.08 (0.51, 2.29)	1.19 (0.55, 2.57)
SO_2	Moderate vs. Slow	P10	0.92 (0.76, 1.12)	0.93 (0.76, 1.13)
SO_2	Moderate vs. Slow	P25	0.97 (0.81, 1.17)	0.98 (0.82, 1.18)
SO_2	Moderate vs. Slow	P75	1.00 (0.84, 1.21)	1.00 (0.83, 1.21)
SO_2	Moderate vs. Slow	P90	1.03 (0.85, 1.24)	1.01 (0.83, 1.23)
SO_2	Accelerated vs. Slow	P10	0.77 (0.36, 1.62)	0.77 (0.37, 1.62)
SO_2	Accelerated vs. Slow	P25	0.91 (0.44, 1.91)	0.91 (0.44, 1.92)
SO_2	Accelerated vs. Slow	P75	1.02 (0.48, 2.14)	1.02 (0.48, 2.14)
SO_2	Accelerated vs. Slow	P90	1.09 (0.51, 2.32)	1.09 (0.51, 2.32)

Pollutant	Comparison	Percentile	GLMM	GAMM
O_3	Moderate vs. Slow	P10	0.95 (0.78, 1.16)	0.99 (0.81, 1.22)
O_3	Moderate vs. Slow	P25	0.97 (0.80, 1.17)	0.99 (0.81, 1.20)
O_3	Moderate vs. Slow	P75	1.00 (0.82, 1.22)	1.00 (0.82, 1.23)
O_3	Moderate vs. Slow	P90	1.01 (0.82, 1.24)	1.03 (0.83, 1.27)
O_3	Accelerated vs. Slow	P10	0.74 (0.35, 1.57)	0.83 (0.39, 1.77)
O_3	Accelerated vs. Slow	P25	0.80 (0.38, 1.70)	0.88 (0.42, 1.84)
O_3	Accelerated vs. Slow	P75	1.00 (0.47, 2.14)	1.00 (0.46, 2.16)
O_3	Accelerated vs. Slow	P90	1.06 (0.49, 2.30)	1.12 (0.52, 2.45)
CO	Moderate vs. Slow	P10	0.94 (0.76, 1.16)	0.94 (0.76, 1.16)
CO	Moderate vs. Slow	P25	0.97 (0.81, 1.17)	0.97 (0.81, 1.17)
CO	Moderate vs. Slow	P75	1.01 (0.83, 1.23)	1.01 (0.83, 1.23)
CO	Moderate vs. Slow	P90	1.01 (0.83, 1.23)	1.01 (0.83, 1.23)
CO	Accelerated vs. Slow	P10	0.94 (0.44, 2.04)	0.94 (0.44, 2.04)
CO	Accelerated vs. Slow	P25	0.97 (0.45, 2.09)	0.97 (0.45, 2.09)
CO	Accelerated vs. Slow	P75	1.01 (0.46, 2.23)	1.01 (0.46, 2.23)
CO	Accelerated vs. Slow	P90	1.01 (0.46, 2.23)	1.01 (0.46, 2.23)

Abbreviations: GLMM, generalized linear mixed model; GAMM, generalized additive mixed model; OR, odds ratio; CI, confidence interval.

This table presents adjusted percentile-based contrasts for the “moderate aging” and “accelerated aging” trajectories, each relative to the “slow aging” trajectory. For the GAMM, ORs compare the estimated odds at each specified exposure percentile with those at the median (P50). For the GLMM, expected ORs were calculated by applying the primary linear model coefficient to the corresponding standardized exposure difference between the specified percentile and P50. Thus, both models were evaluated over the same exposure contrast. The 95% CIs reflect uncertainty in these specific point-to-point contrasts and may be wider than CIs for the overall linear association. The purpose of this comparison was to evaluate whether the nonlinear estimates materially deviated from the corresponding linear expectation.

Table S16. Comparison of unadjusted and covariate-adjusted associations between three-year mean environmental exposures and biological aging trajectories.

Exposure	Comparison	Unadjusted OR (95% CI)	Covariate-adjusted OR (95% CI)
**Green space**			
NDVI 250 m	Moderate vs. Slow	0.999 (0.945, 1.055)	0.996 (0.940, 1.056)
	Accelerated vs. Slow	0.890 (0.797, 0.994)	0.866 (0.772, 0.971)
NDVI 500 m	Moderate vs. Slow	0.997 (0.941, 1.055)	0.996 (0.938, 1.058)
	Accelerated vs. Slow	0.869 (0.774, 0.976)	0.844 (0.750, 0.955)
NDVI 750 m	Moderate vs. Slow	0.996 (0.942, 1.054)	0.998 (0.941, 1.059)
	Accelerated vs. Slow	0.877 (0.781, 0.985)	0.857 (0.759, 0.967)
NDVI 1,000 m	Moderate vs. Slow	0.994 (0.939, 1.053)	0.996 (0.938, 1.058)
	Accelerated vs. Slow	0.885 (0.786, 0.997)	0.868 (0.766, 0.982)
**Air pollutants**			
$PM_{2.5}$	Moderate vs. Slow	1.038 (0.990, 1.088)	1.045 (0.995, 1.097)
	Accelerated vs. Slow	1.104 (1.030, 1.184)	1.127 (1.049, 1.210)
PM_{10}	Moderate vs. Slow	1.067 (1.023, 1.113)	1.063 (1.019, 1.110)
	Accelerated vs. Slow	1.106 (1.044, 1.171)	1.127 (1.063, 1.196)
NO_2	Moderate vs. Slow	1.054 (1.007, 1.104)	1.053 (1.004, 1.105)
	Accelerated vs. Slow	1.047 (0.968, 1.133)	1.061 (0.978, 1.151)
SO_2	Moderate vs. Slow	1.027 (0.978, 1.079)	1.043 (0.991, 1.098)
	Accelerated vs. Slow	1.119 (1.026, 1.221)	1.149 (1.049, 1.258)
O_3	Moderate vs. Slow	0.998 (0.945, 1.054)	1.022 (0.965, 1.083)
	Accelerated vs. Slow	1.127 (1.017, 1.250)	1.152 (1.034, 1.283)
CO	Moderate vs. Slow	1.026 (0.966, 1.090)	1.028 (0.965, 1.096)
	Accelerated vs. Slow	1.024 (0.910, 1.153)	1.026 (0.906, 1.162)

Abbreviations: OR, odds ratio; CI, confidence interval.

Odds ratios (ORs) and 95% confidence intervals (CIs) were estimated using separate multinomial logistic mixed-effects models for each exposure, with the “slow aging” trajectory serving as the reference group. Estimates represent the odds of membership in the “moderate aging” or “accelerated aging” trajectory associated with a 1-standard deviation (SD) increase in exposure. Exposure variables were standardized using the standard deviation of the analytical sample. Unadjusted models included only the exposure and a random intercept for workplace, without any fixed-effect covariates. Covariate-adjusted models additionally included sex, baseline age, smoking status, alcohol consumption, prevalent chronic diseases, and individual-level socioeconomic status. The unadjusted and covariate-adjusted models used the same analytical sample, outcome coding, exposure standardization, and workplace random-effect structure. Environmental exposures were estimated using geocoded workplace addresses, averaged over three years, and assigned to participants according to their workplaces.

Table S17. Gaussian-mixture exposure components and component-specific associations of $PM_{2.5}$ and PM_{10} with biological aging trajectories.

Panel A. Participant distribution across exposure components

Exposure	Component	Participants, n (%)	Slow aging, n (%)	Moderate aging, n (%)	Accelerated aging, n (%)
$PM_{2.5}$	Lower	11,053 (94.0)	3,613 (32.7)	5,509 (49.8)	1,931 (17.5)
	Higher	707 (6.0)	219 (31.0)	366 (51.8)	122 (17.2)
PM_{10}	Lower	8,172 (69.5)	2,763 (33.8)	4,042 (49.5)	1,367 (16.7)
	Higher	3,588 (30.5)	1,069 (29.8)	1,833 (51.1)	686 (19.1)

Panel B. Associations within exposure components and tests of differences in slopes

Exposure	Comparison	Component	OR per 1 SD (95% CI)	*P* value	*P* for interaction
$PM_{2.5}$	Moderate vs. slow	Lower	1.049 (0.987, 1.115)	0.127	0.681
		Higher	—*	—*	
	Accelerated vs. slow	Lower	1.170 (1.072, 1.278)	<0.001	0.731
		Higher	—*	—*	
PM_{10}	Moderate vs. slow	Lower	0.808 (0.639, 1.021)	0.075	0.745
		Higher	0.957 (0.537, 1.708)	0.883	
	Accelerated vs. slow	Lower	0.650 (0.453, 0.933)	0.019	0.261
		Higher	1.277 (0.548, 2.974)	0.571	

Abbreviations: CI, confidence interval; ICL, integrated complete-data likelihood; OR, odds ratio; SD, standard deviation.

$PM_{2.5}$ and PM_{10} components were identified separately using Gaussian mixture models applied to 521 distinct exposure profiles with equal weighting, thereby preventing repeated assignments of the same workplace-based exposure profiles from dominating component identification. Models with one to five components and equal- or unequal-variance specifications were evaluated, and the model with the highest integrated complete-data likelihood (ICL) was selected; a two-component unequal-variance model was selected for each pollutant. Participants were assigned to components according to maximum posterior probability. Analyses using the original concentrations and standardized values produced virtually identical classifications. Within-component odds ratios (ORs) and 95% confidence intervals (CIs) were estimated using separate covariate-adjusted multinomial logistic mixed-effects models, with the slow aging trajectory as the reference group and a random intercept for workplace. Models were adjusted for sex, baseline age, smoking status, alcohol consumption, prevalent chronic diseases, and individual-level socioeconomic status. ORs represent differences in the odds of trajectory-group membership per 1-standard deviation (SD) increase in exposure, using the corresponding full-sample SD to maintain a common scale across components. Because exposure variation was limited within each component, all component-specific per-SD estimates should be interpreted cautiously. Exposure-by-component interaction terms in full-sample models were used to test whether the linear slopes differed between components for each trajectory comparison. Percentages in Panel A were calculated within each component.

*The independently fitted model for the higher $PM_{2.5}$ component was not reliably estimable because of limited within-component exposure variation and sparse outcome–covariate combinations.

Supplementary Figures

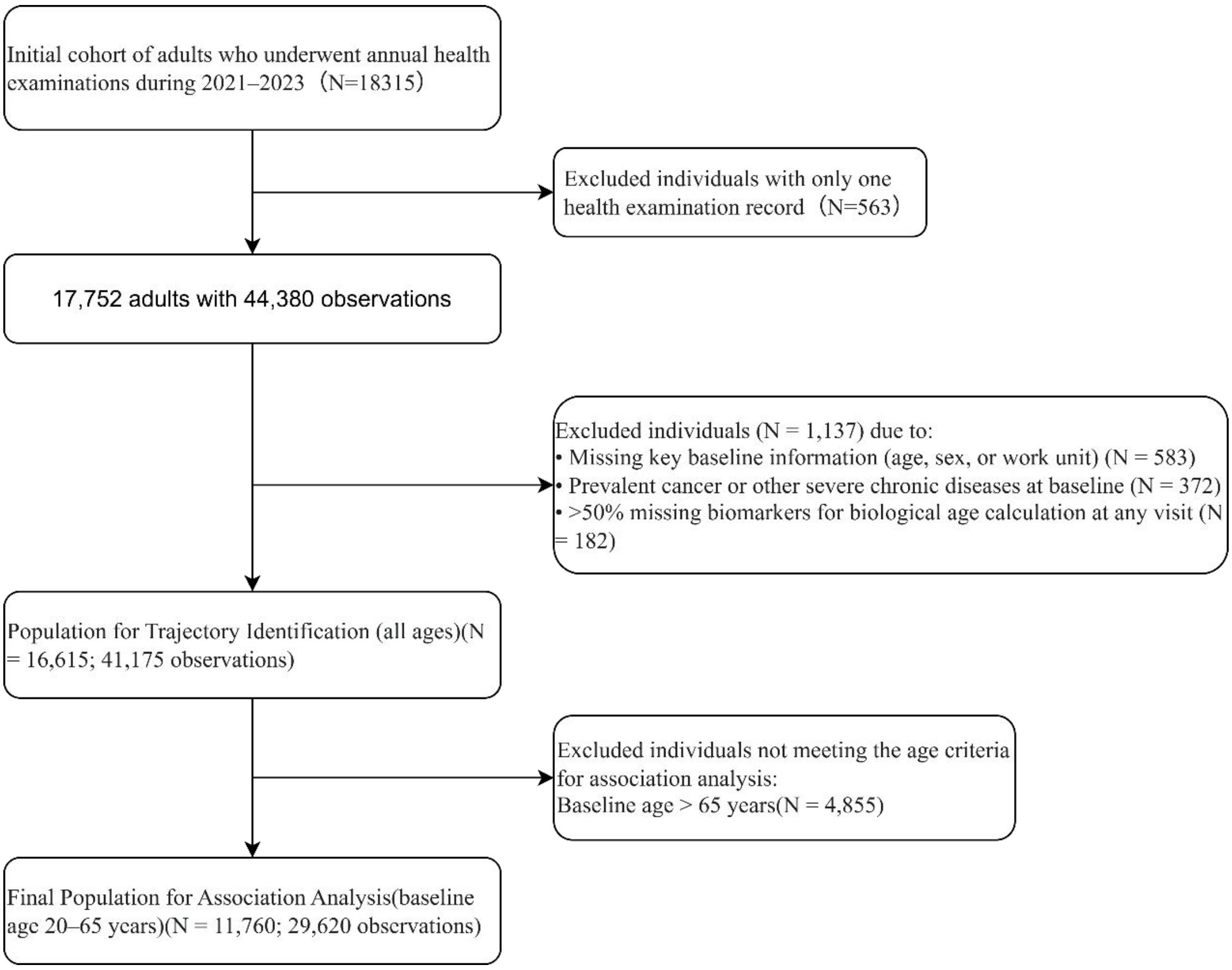

Figure S1. Flow diagram of participant selection.

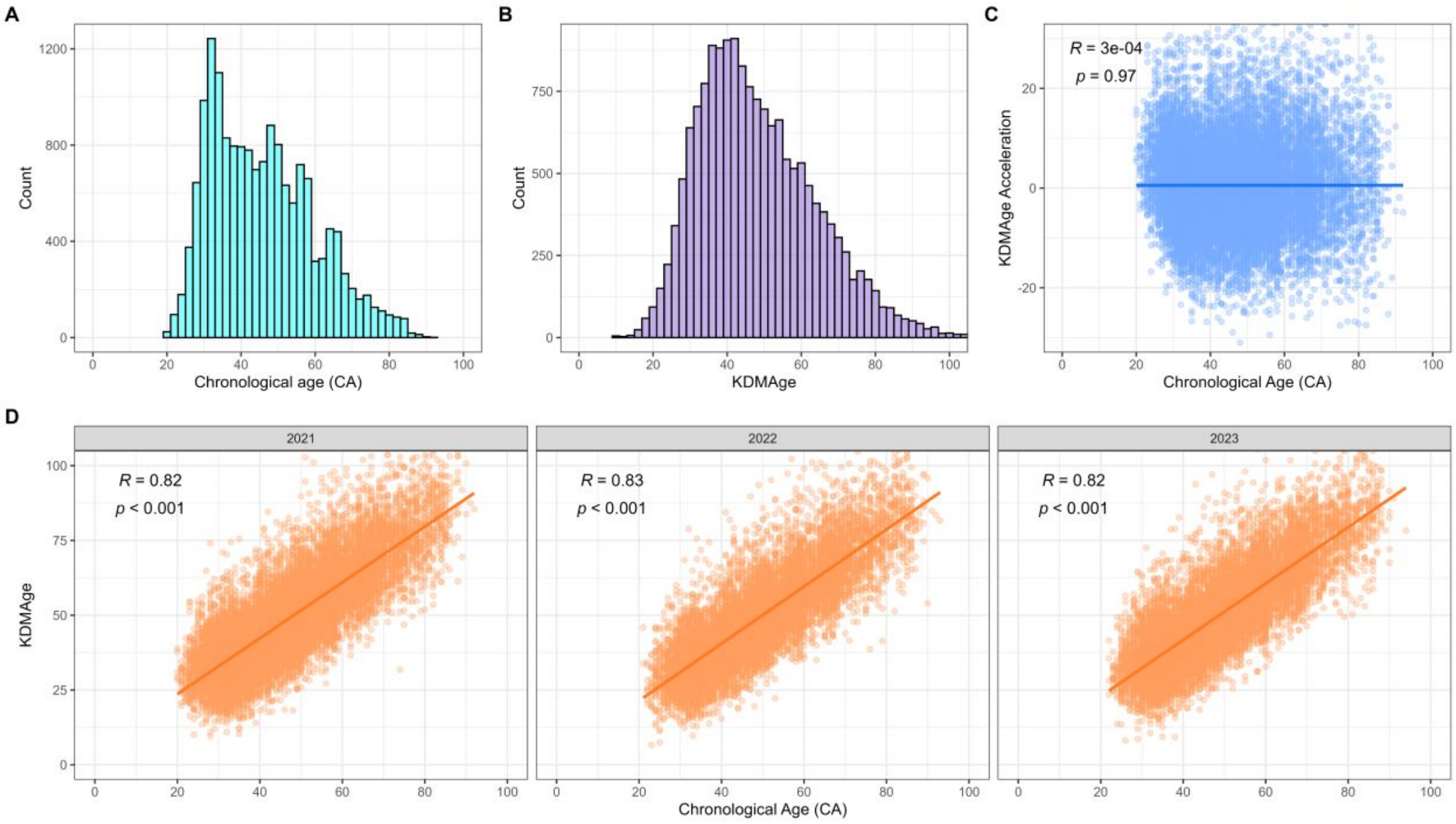

Figure S2. Distribution and correlation of chronological and biological ages.

(A, B) Histograms showing the distribution of Chronological Age (CA) and Klemera-Doubal Method Biological Age (KDM-BA) in the study population at baseline. (C) Scatter plot of Biological Age Acceleration (BAA) against CA at baseline. (D) Scatter plot of KDM-BA against CA across different time points (visits).

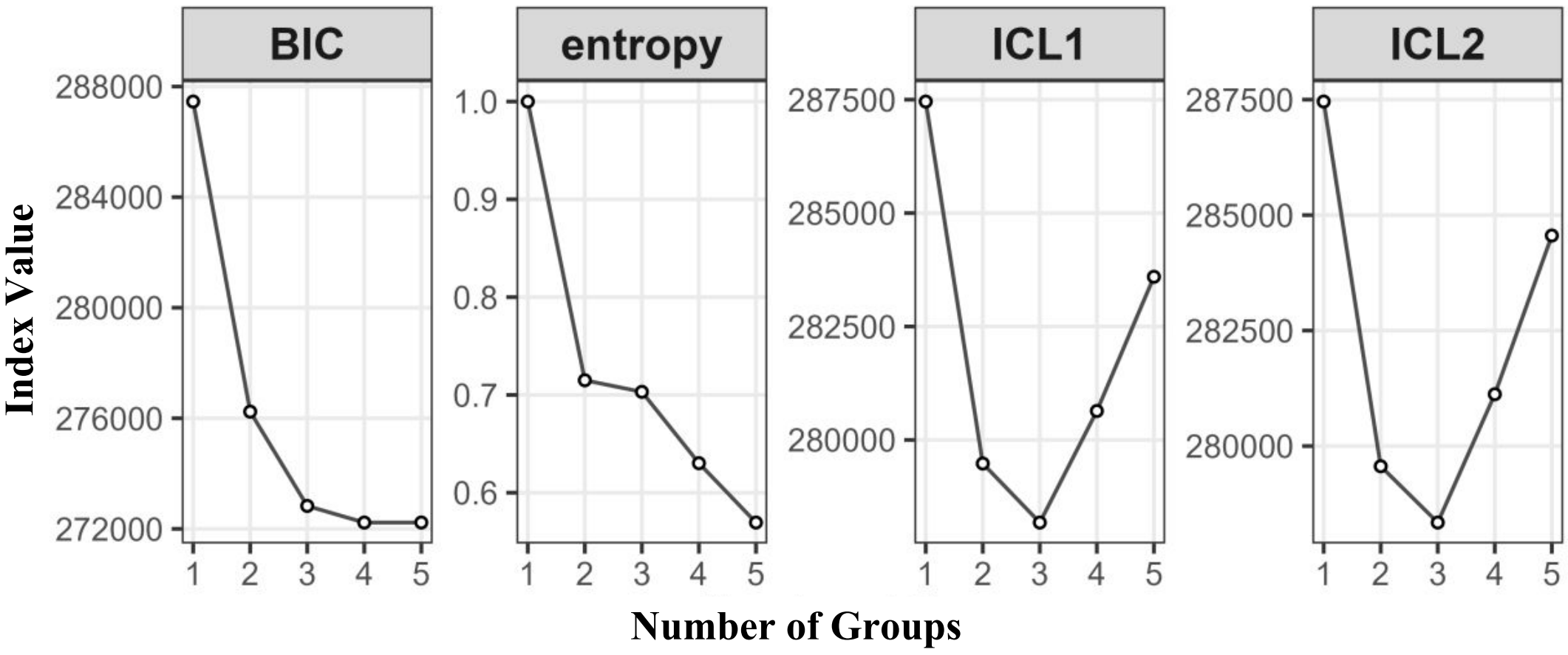

Figure S3. Model fit and classification quality indices for group-based trajectory models (GBTM) with different numbers of latent trajectory groups.

The figure displays four key indices used to determine the optimal number of trajectory groups (from 1 to 5). (A) The Bayesian Information Criterion (BIC), where a lower value indicates better model fit. (B) The Entropy, which measures classification certainty (closer to 1 is better). (C, D) The Integrated Completed Likelihood (ICL) criterion, which penalizes for poor classification (lower is better). Based on these indices, the three-group model was selected as the optimal solution. While the BIC continued to decrease slightly for the four-group model, the "elbow" of the BIC curve appears at three groups. More importantly, the ICL criterion reached its minimum value at three groups, and the entropy for the three-group model (0.703) was deemed acceptable.

Abbreviations: BIC, Bayesian Information Criterion; ICL, Integrated Completed Likelihood.

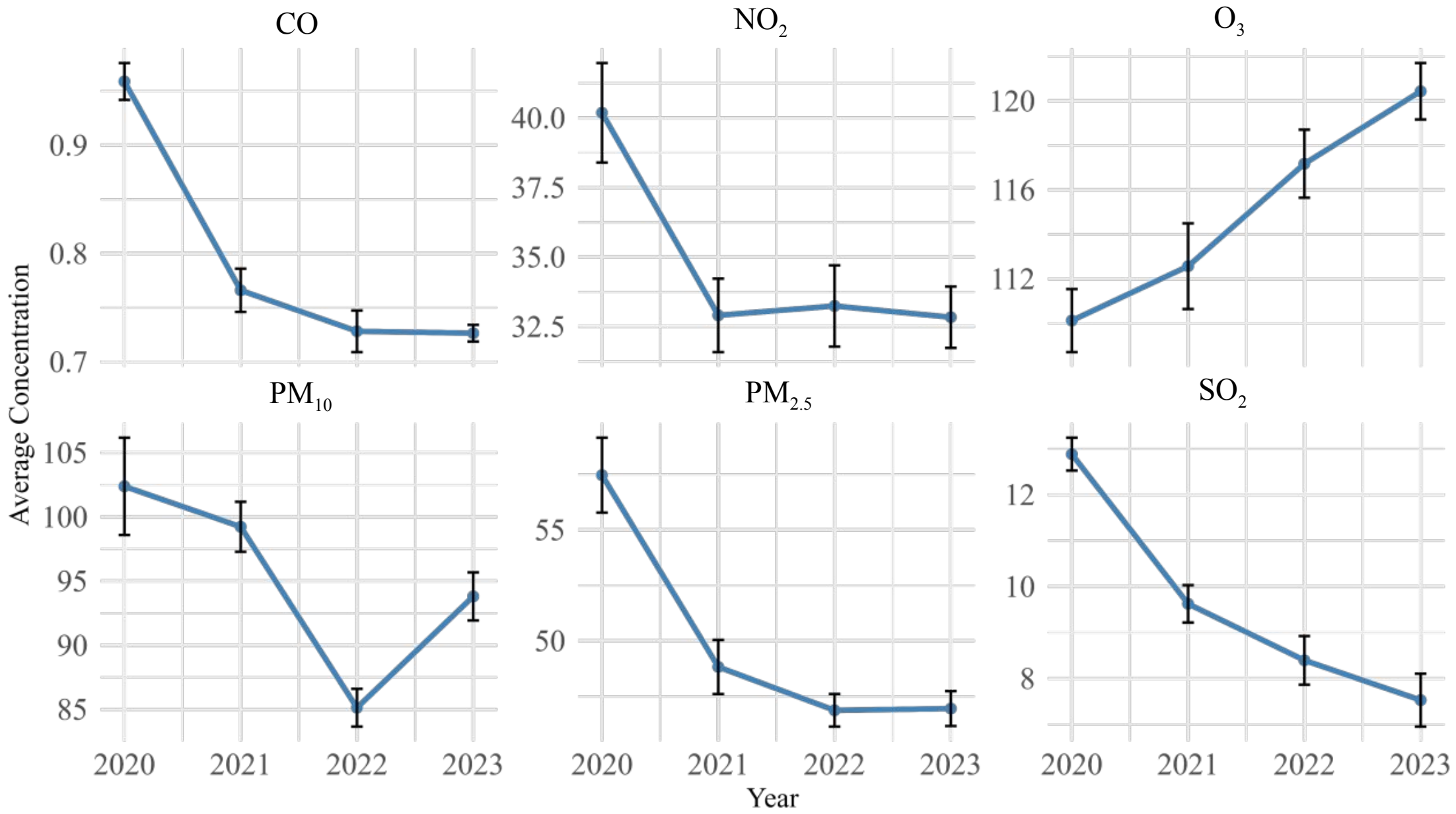

Figure S4. Temporal trends of annual average air pollutant concentrations in the study area (2020–2023).

The figure displays the annual variations in the concentrations of six major air pollutants (CO, NO_2, O_3, PM_{10}, $PM_{2.5}$, and SO_2) assigned to participants' workplace addresses. Data points represent the mean concentration for each year, and error bars indicate the standard deviation (SD). Note the increasing trend for ozone (O_3) in contrast to the decreasing trends for other pollutants.

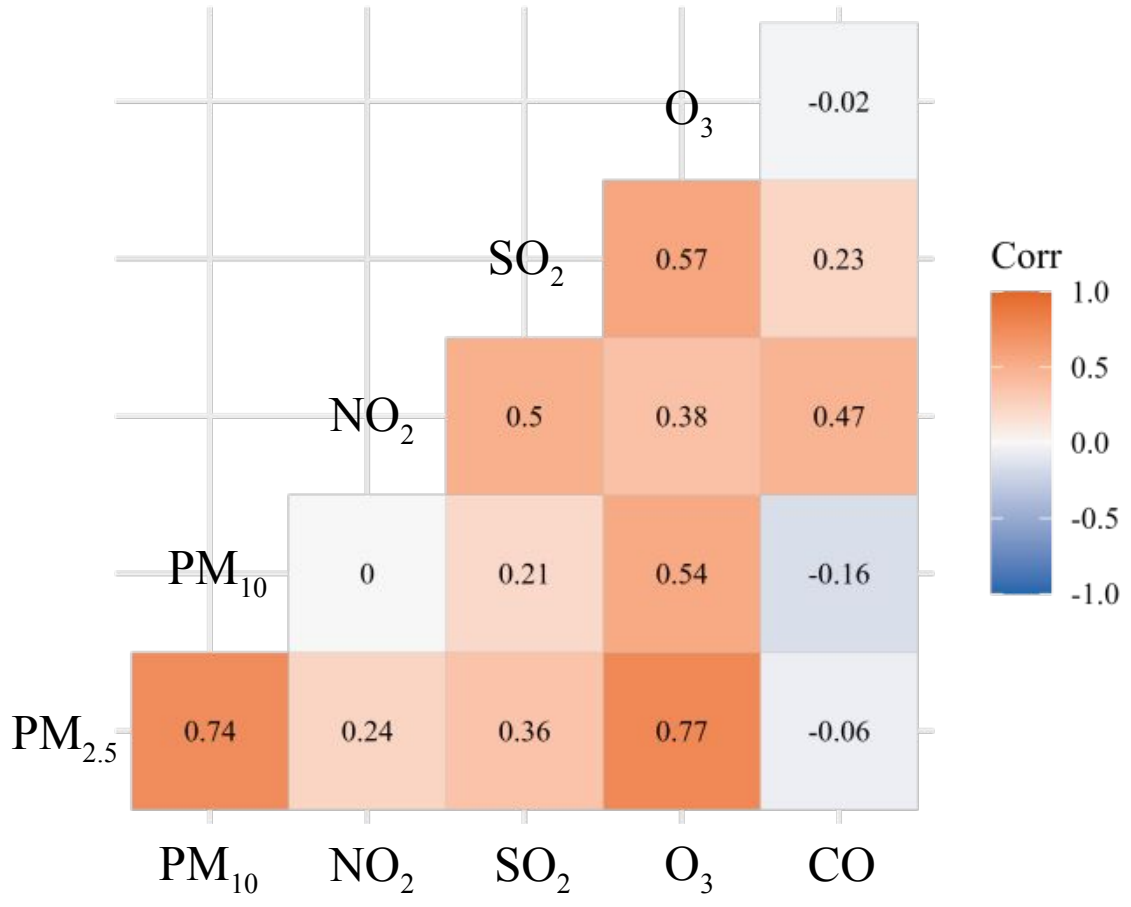

Figure S5. Spearman's correlation coefficients among long-term air pollutants (calculated at the workplace level).

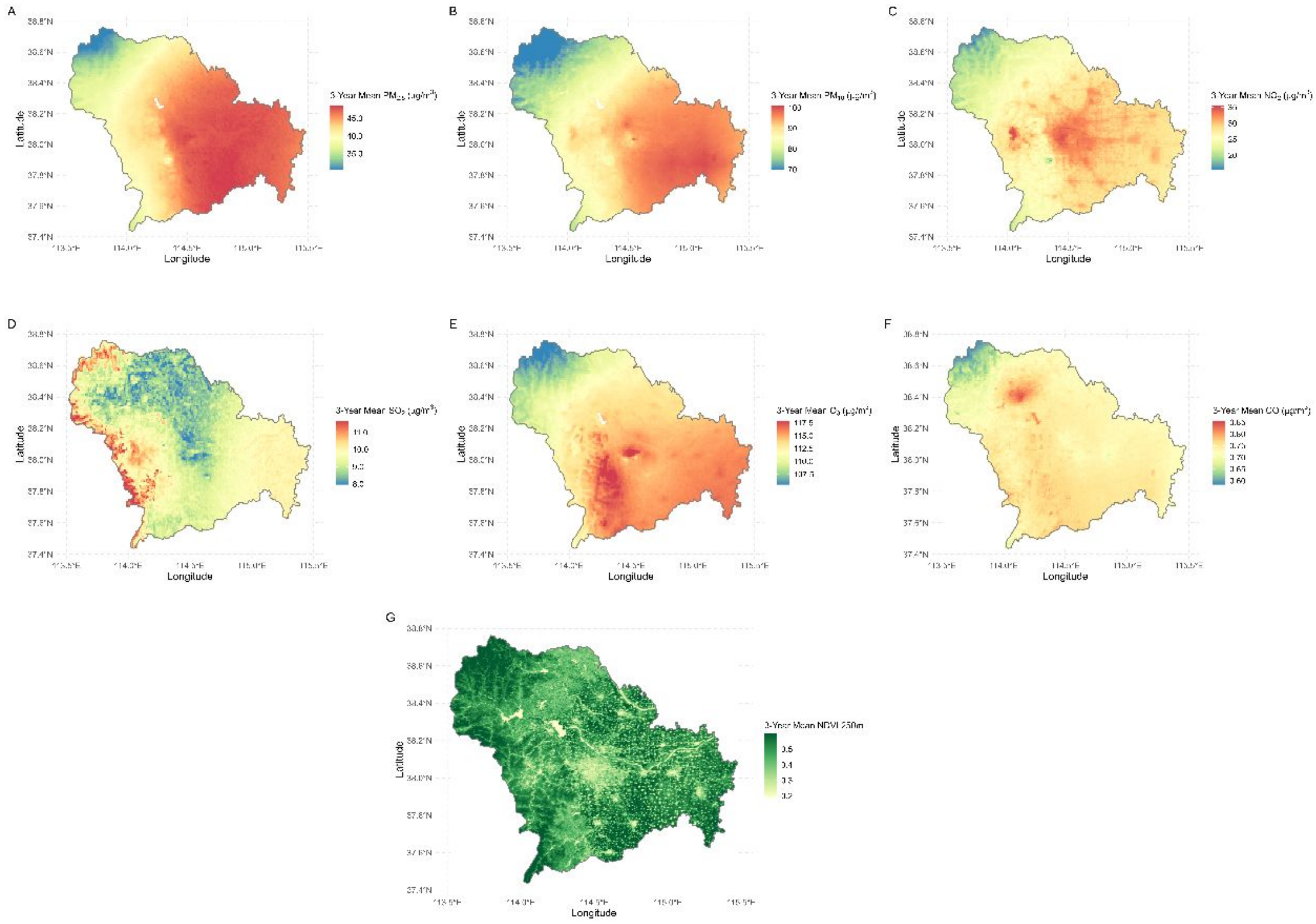

Figure S6. High-resolution spatial distribution of 3-year mean environmental exposures across the study area.

The maps illustrate the continuous intra-urban gradients of ambient air pollutants and green space from 2021 to 2023. Panels (A) to (F) display the 1-km resolution spatial distribution of $PM_{2.5}$, PM_{10}, NO_2, SO_2, O_3, and CO, respectively, derived from the ChinaHighAirPollutants (CHAP) dataset. Panel (G) presents the 250-m resolution Normalized Difference Vegetation Index (NDVI). Exact participant coordinates are omitted to strictly comply with ethical and confidentiality agreements.

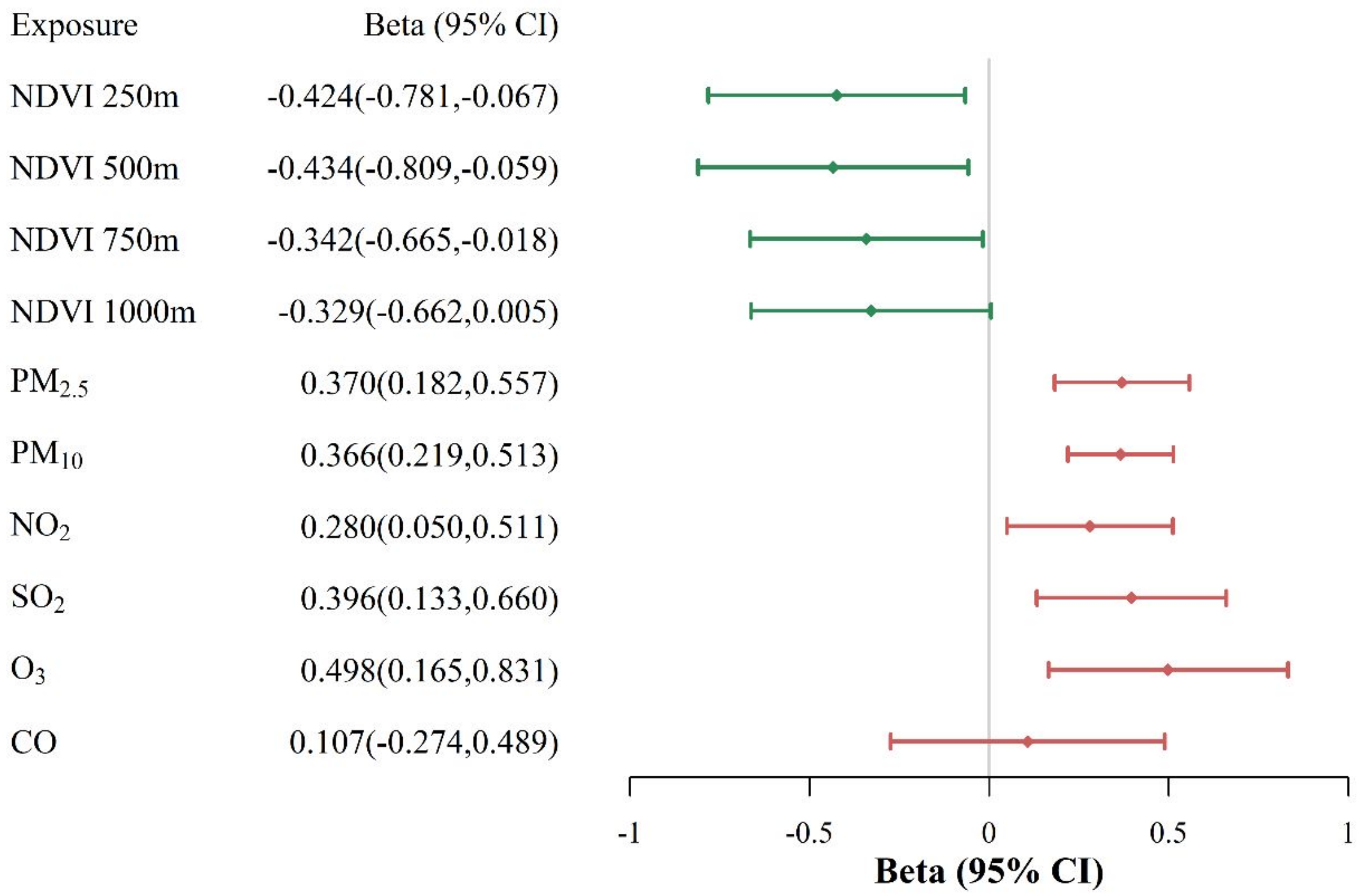

Figure S7. Associations between long-term environmental exposures and continuous biological age acceleration (BAA) derived from linear mixed models.
The figure displays the beta coefficients (*β*) and 95% confidence intervals (CIs) representing the change in biological age acceleration (BAA, in years) associated with a one standard deviation (SD) increase in each environmental exposure. Models were fitted using Linear Mixed Models (LMM) to account for the repeated measures and hierarchical data structure. All models were adjusted for sex, baseline age, smoking status, alcohol consumption, history of chronic diseases, and individual-level socioeconomic status. Random intercepts were included for both workplace and individual ID.
Interpretation: Values less than 0 indicate a protective association (decelerated aging), while values greater than 0 indicate an adverse association (accelerated aging).

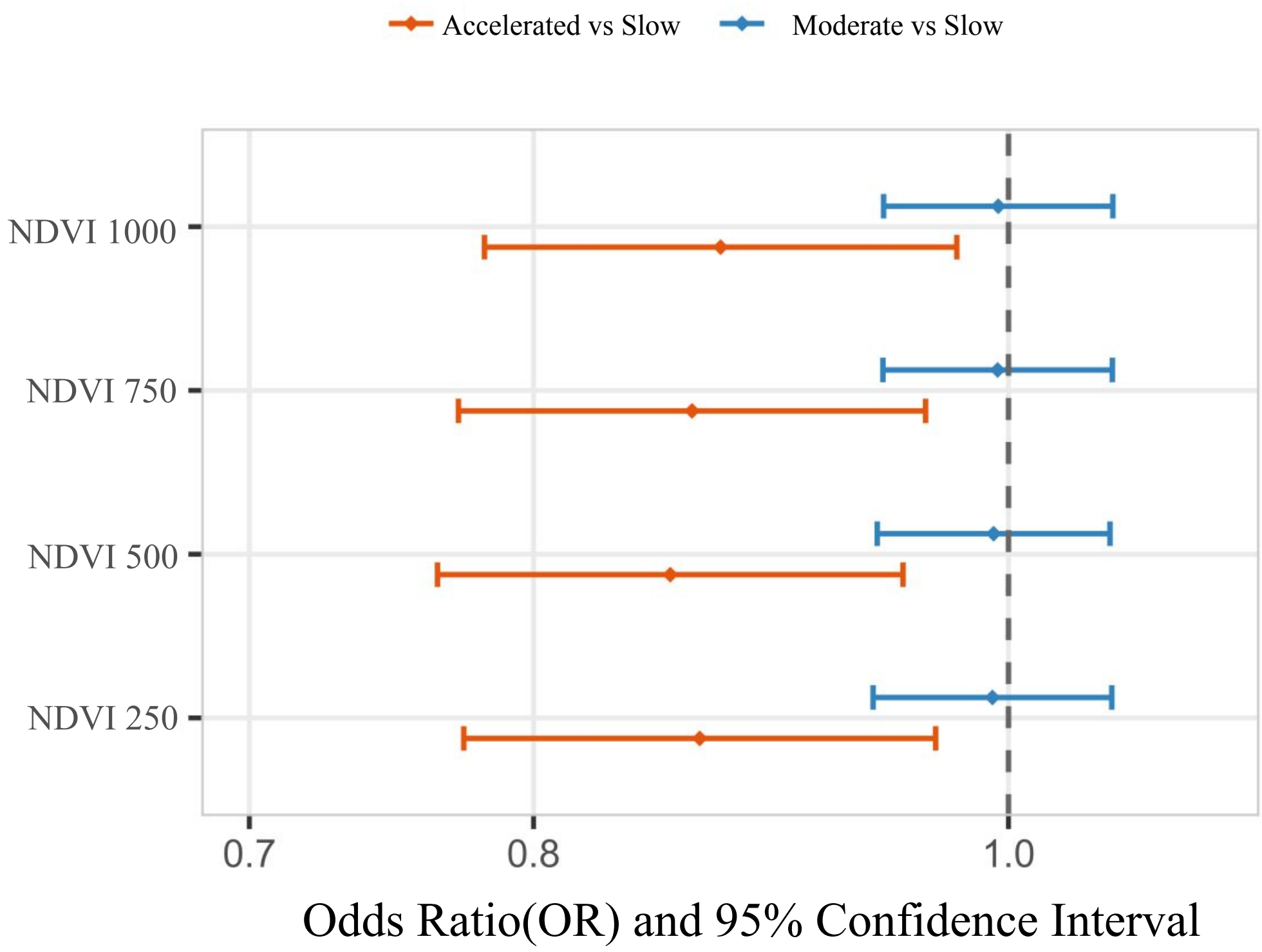

Figure S8. Sensitivity analysis using a 1-year lagged exposure window (2020–2022) for green space exposures.

The forest plot displays the odds ratios (ORs) and 95% confidence intervals (CIs) for the association between green space exposure (NDVI) and aging trajectories, using exposure data averaged over the 2020–2022 period (1-year lag). The results show the associations for the "Moderate vs. Slow" and "Accelerated vs. Slow" aging trajectory comparisons across different buffer sizes. The direction and magnitude of these associations are consistent with the primary analysis (2021–2023 exposure) presented in Figure 2 of the main text. All models were fully adjusted.

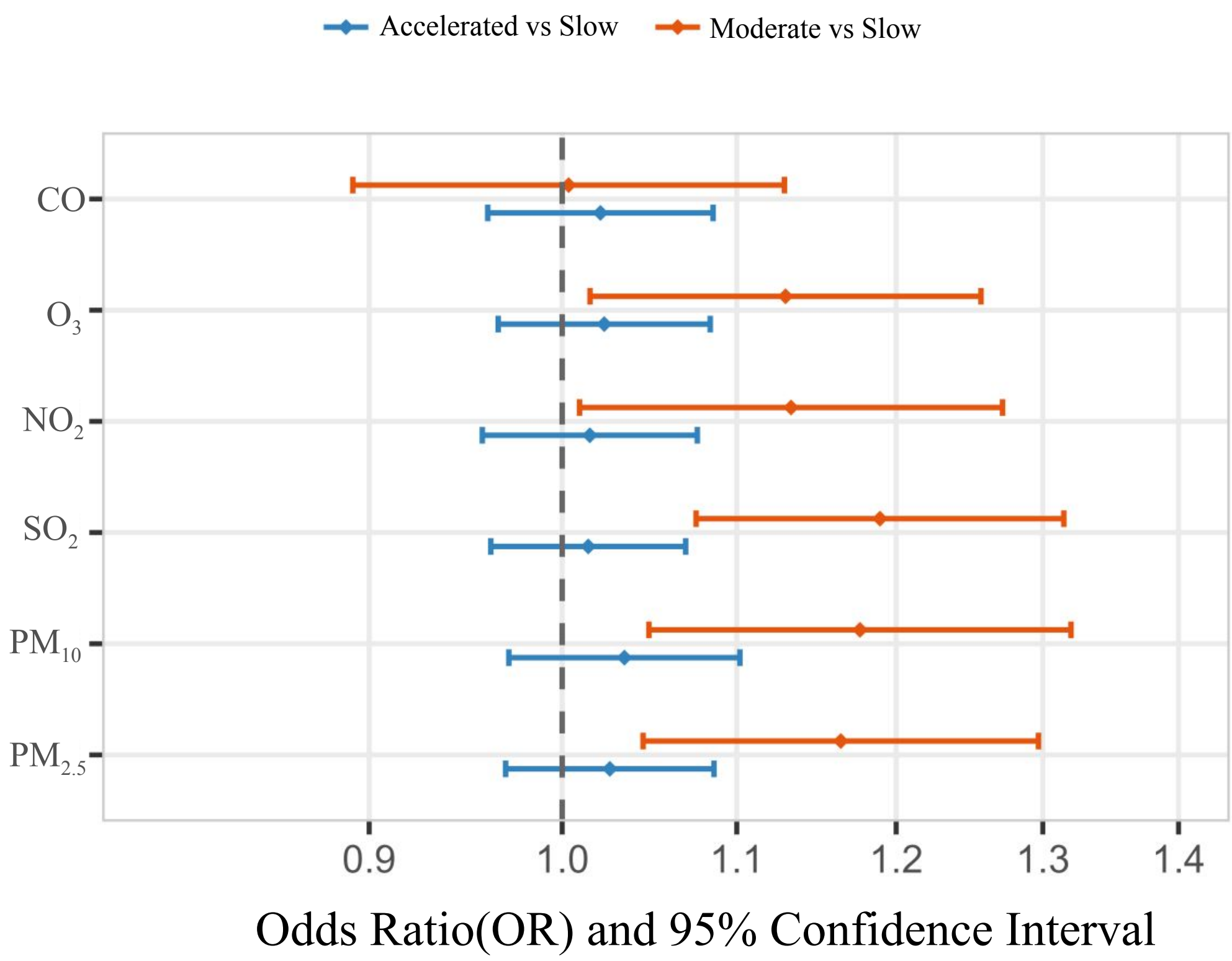

Figure S9. Sensitivity analysis using a 1-year lagged exposure window (2020–2022) for air pollution exposures.

The forest plot displays the odds ratios (ORs) and 95% confidence intervals (CIs) for the association between single air pollutants and aging trajectories, using exposure data averaged over the 2020–2022 period (1-year lag). The results show the associations for the "Moderate vs. Slow" and "Accelerated vs. Slow" aging trajectory comparisons across different buffer sizes. The direction and magnitude of these associations are consistent with the primary analysis (2021–2023 exposure) presented in Figure 2 of the main text. All models were fully adjusted.

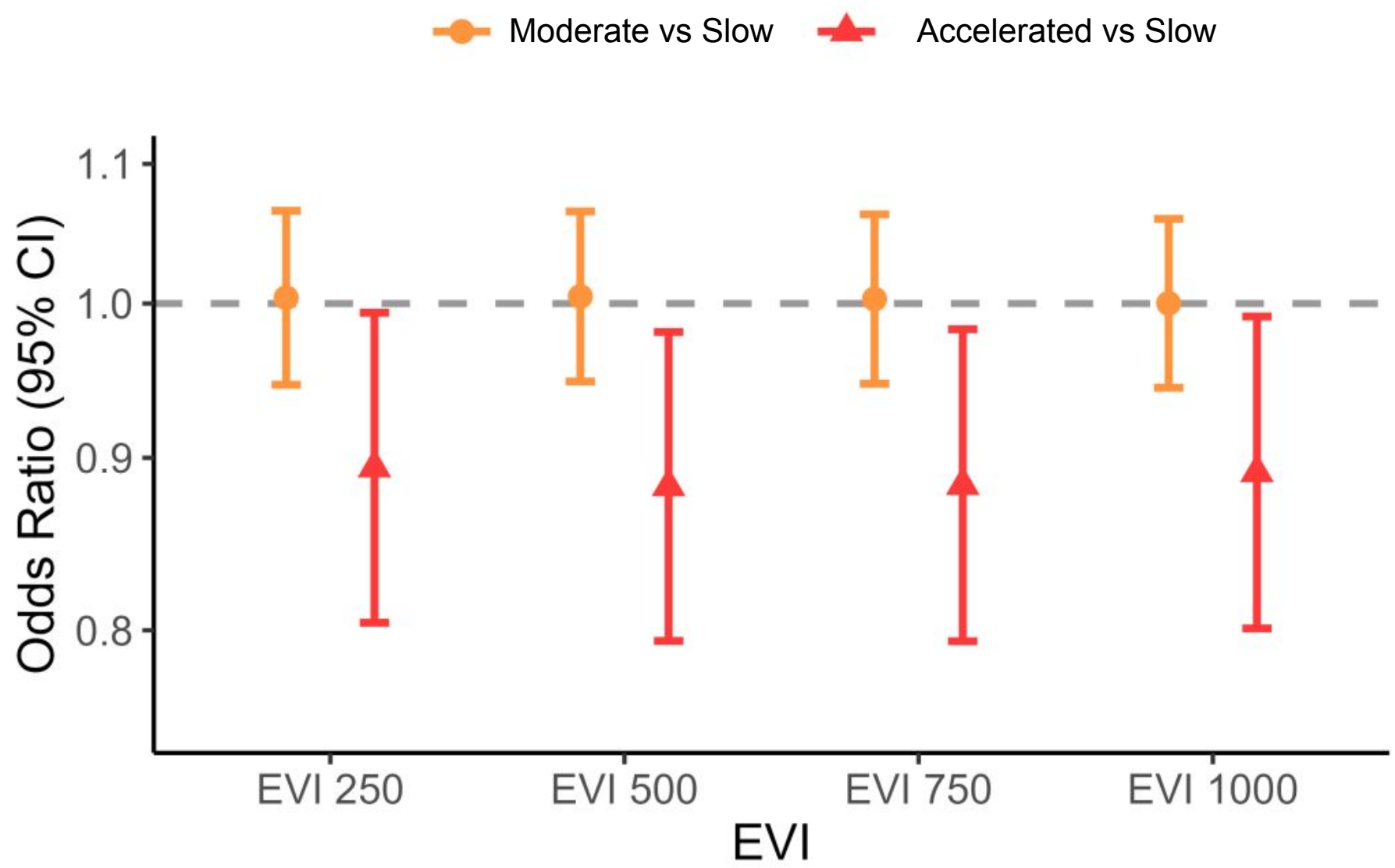

Figure S10. Sensitivity analysis for the associations between the Enhanced Vegetation Index (EVI) at multiple buffer sizes and biological aging trajectories.

The forest plot presents the odds ratios (ORs) and 95% confidence intervals (CIs) for being in the "moderate aging" or "accelerated aging" trajectory, compared to the "slow aging" trajectory, associated with a one-standard-deviation (1-SD) increase in EVI. Results are shown across four spatial scales (250m, 500m, 750m, and 1000m buffers). All models were fully adjusted.

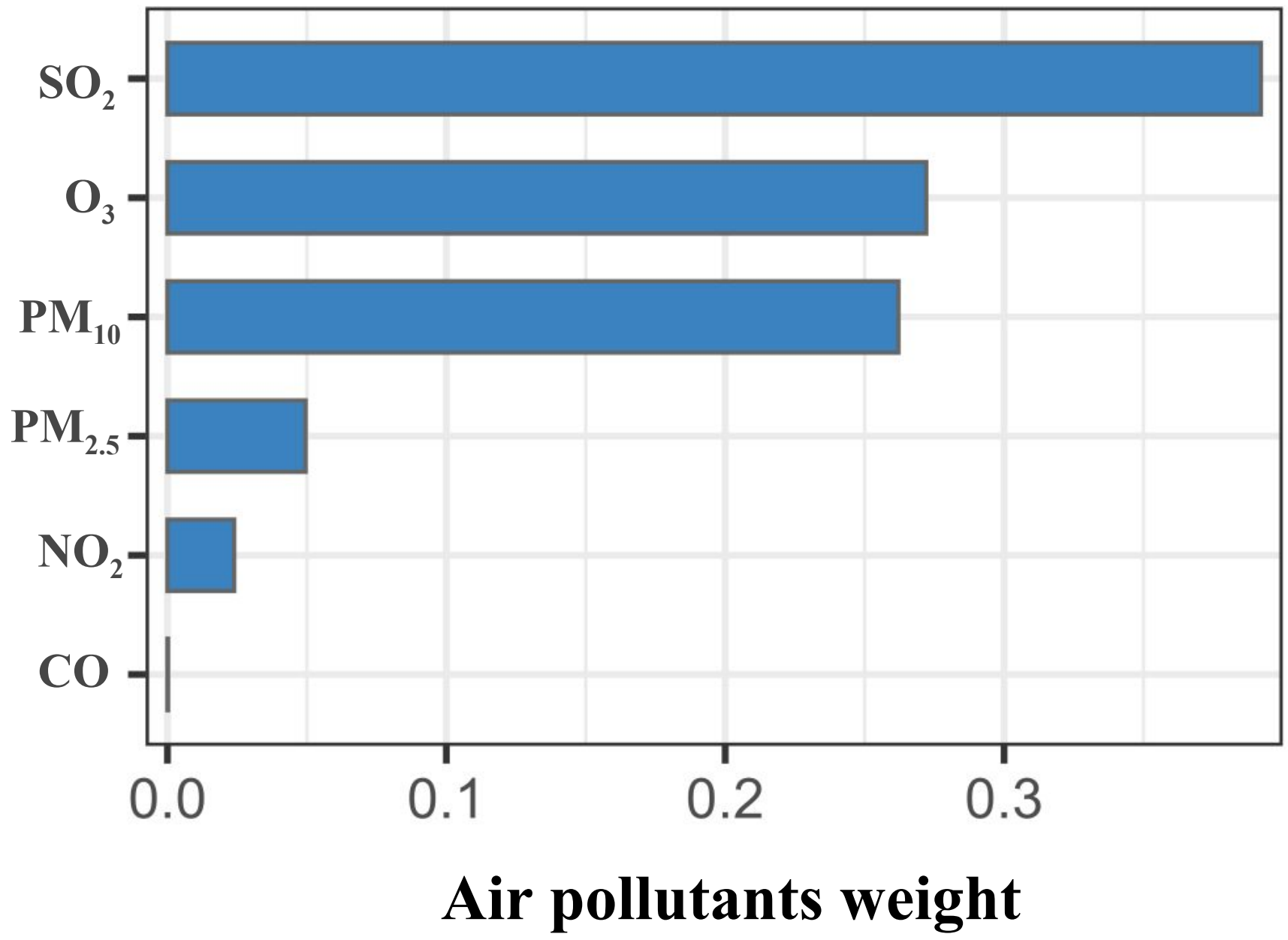

Figure S11. Pollutant weights from the Weighted Quantile Sum (WQS) regression model with 10 quantiles.

The bar plot displays the weights of individual air pollutants for the association with the binary outcome of "Accelerated Aging," derived from a WQS model using deciles (q=10) instead of quartiles (q=4). The pattern of weights is highly consistent with the primary analysis (q=4).

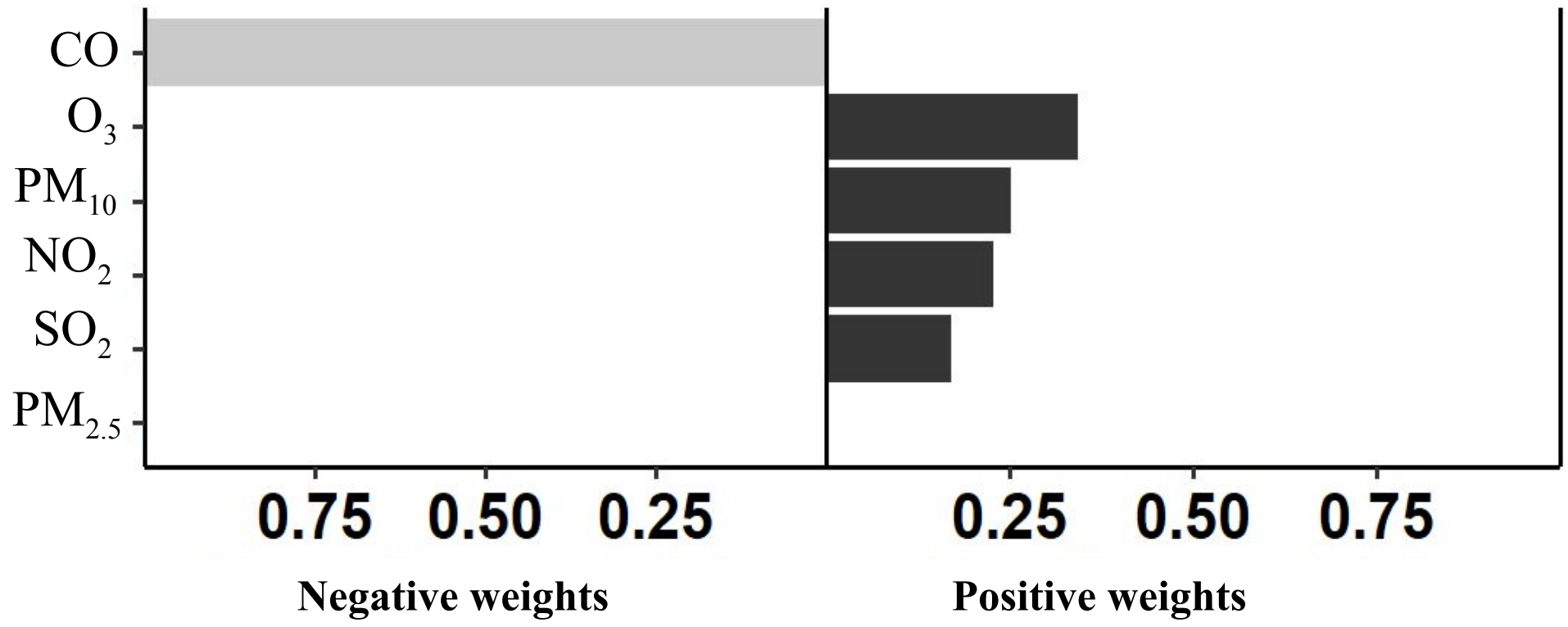

Figure S12. Pollutant weights from the Quantile G-Computation (QGC) model.

The butterfly plot shows the positive (dark bars) and negative (light bars) weights of individual air pollutants for the association with the binary outcome of "Accelerated Aging," derived from the QGC sensitivity analysis. Consistent with the primary WQS analysis, PM_{10} and O_3 show substantial positive contributions to the overall mixture effect.

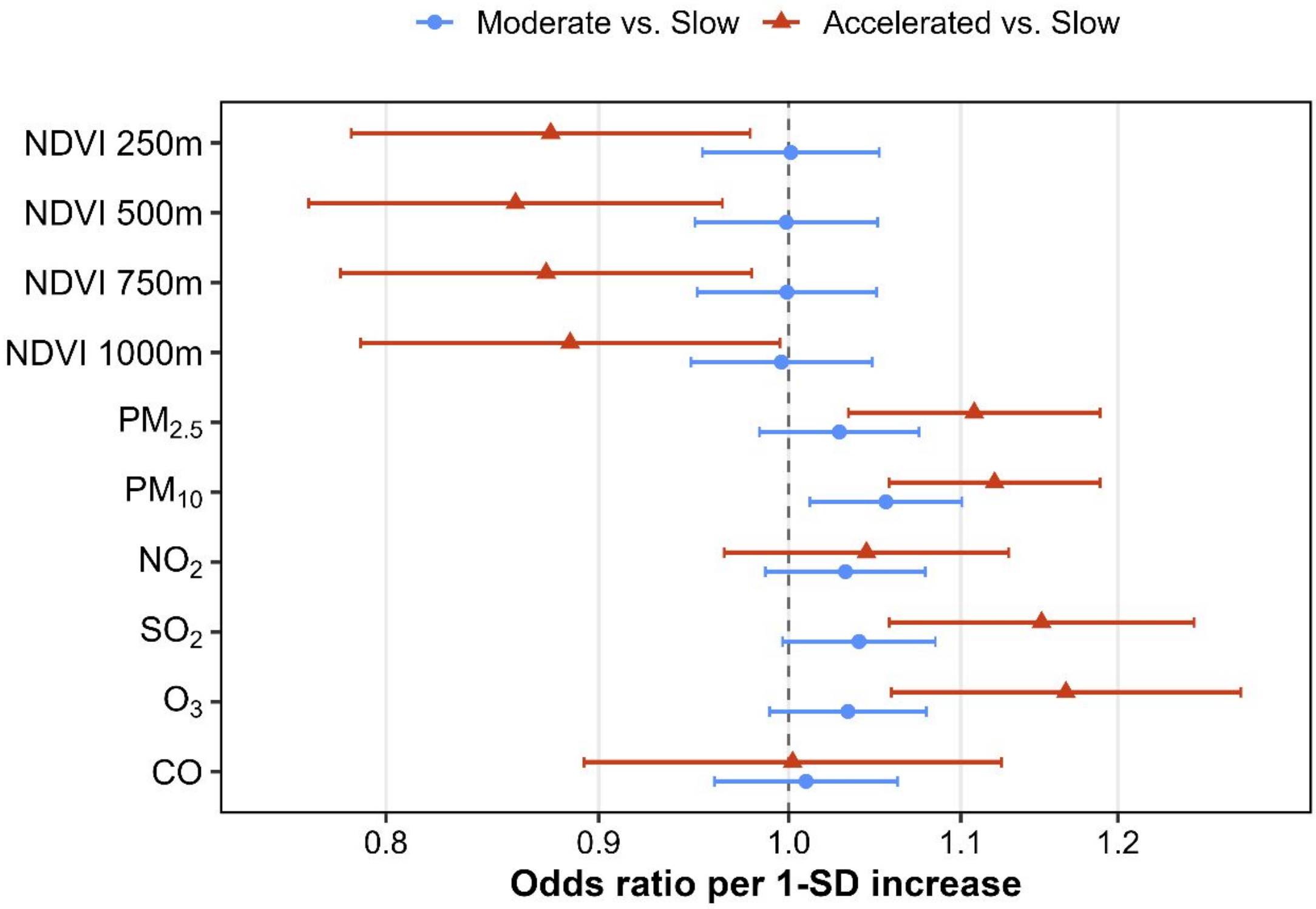

Figure S13. Associations of green space and air pollutants with aging trajectories after additional adjustment for occupational categories.

To address potential residual confounding by occupational characteristics, participants were classified into five broad occupational categories based on their basic individual-level occupational information (e.g., civil servants, physicians, teachers, clerical staff, and service workers, etc.) in conjunction with workplace unit names: public administration/community services, education/research/health professions, financial services, enterprise/utility categories, and service/support/other occupations. Odds ratios (ORs) and 95% confidence intervals (CIs) are shown for "moderate aging" and "accelerated aging" compared with "slow aging" per 1-SD increase in each standardized exposure. Models were adjusted for the same covariates as the primary models (sex, baseline age, smoking status, alcohol consumption, household income, and prevalent chronic diseases), with additional adjustment for the occupational category and a random intercept for the workplace. The dashed vertical line indicates the null effect (OR = 1.0).

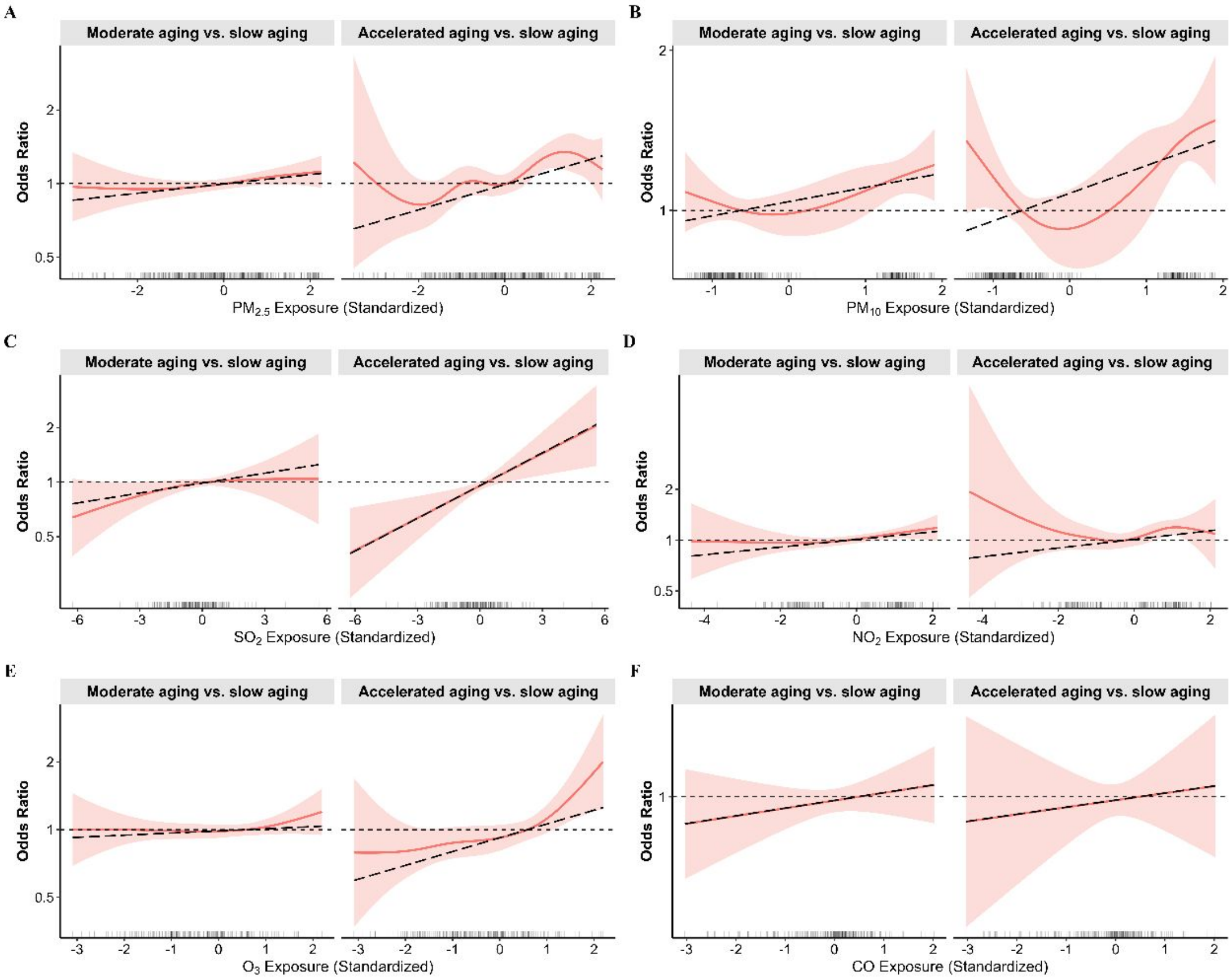

Figure S14. Comparison of generalized additive mixed models (GAMM) and generalized linear mixed models (GLMM) with rug plots.

To assess whether potential deviations from linearity materially altered the interpretation of the primary analyses, GAMM non-linear splines (solid red lines with 95% confidence intervals) were overlaid with the corresponding GLMM linear estimates (dashed black lines). Both curves were centered at the median exposure level (OR = 1.0). Rug plots along the x-axis indicate the distribution of observed exposure values. Overall, the non-linear and linear exposure-response curves showed broadly similar patterns across the ranges where most observations were concentrated, indicating that the primary interpretations based on GLMMs remained robust despite modest departures from linearity in some exposure ranges.
